# Withaferin A suppresses malignant progression of leukemia by reducing promoter methylation levels

**DOI:** 10.1038/s41420-026-03435-x

**Published:** 2026-11-13

**Authors:** Dongxiao Li, Mohamed-Elamir F. Hegazy, Mohamed Elbadawi, Chunmei Jin, Paola B. Arimondo, Xiao Lei, Sabine M. Klauck, A. A. Leslie Gunatilaka, Gerhard Bringmann, Thomas Efferth, Mona Dawood

**Affiliations:** 1 Department of Pharmaceutical Biology, Institute of Pharmaceutical and Biomedical Sciences, Johannes Gutenberg University, Mainz, 55128 Germany; 2 Chemistry of Medicinal Plants Department, National Research Centre, Giza, 12622 Egypt; 3 Epigenetic Chemical Biology, Institute Pasteur, Université Paris Cité, CNRS UMR3523, Paris, 75724 France; 4 Division of Cancer Genome Research, German Cancer Research Center (DKFZ) Heidelberg, National Center for Tumor Diseases (NCT), NCT Heidelberg, a partnership between DKFZ and University Hospital Heidelberg, Heidelberg, 69120 Germany; 5 Southwest Center for Natural Products Research and Commercialization, School of Natural Resources and the Environment, Tucson, AZ USA; 6 Institute of Organic Chemistry, University of Würzburg, Würzburg, 97074 Germany

**Keywords:** Pharmacology, Mechanism of action, Natural products, Transcriptomics, RNA sequencing

## Abstract

T-cell acute lymphoblastic leukemia (T-ALL) is an aggressive hematologic malignancy with limited effective treatment options. Withaferin A, a steroidal lactone derived from *Withania somnifera*, has demonstrated broad anticancer activity across various tumor models. Emerging evidence suggests its potential to modulate DNA methylation and to suppress malignant behaviors, though the underlying mechanisms have so far remained unclear. In this study, an in-depth investigation of the pharmacological effects of withaferin A in T-ALL was conducted, focusing on its epigenetic regulatory role. Cytotoxicity assays showed that withaferin A exhibited the strongest activity among its analogs, particularly against CCRF-CEM cells. Through the integration of bioinformatics, network pharmacology, and functional cell experiments, it was found that withaferin A induces G2/M cell cycle arrest, promotes apoptosis, inhibits migration, and suppresses microtubule polymerization in T-ALL cells. Luciferase reporter experiments further confirmed that withaferin A decreases methylation of the CMV promoter. Also, withaferin A alters the expression levels of *P21, ITPKB, RAD21, PRR11, AFMID, CCDC34*, and *CYB5R1* in CCRF-CEM cells. In conclusion, our study provides comprehensive evidence that withaferin A suppresses the malignant progression of T-ALL by modulating DNA methylation levels. The study delivers novel evidence for its role in DNA methylation and lays an experimental foundation for its potential clinical application in T-cell acute lymphoblastic leukemia.

**Figure:**
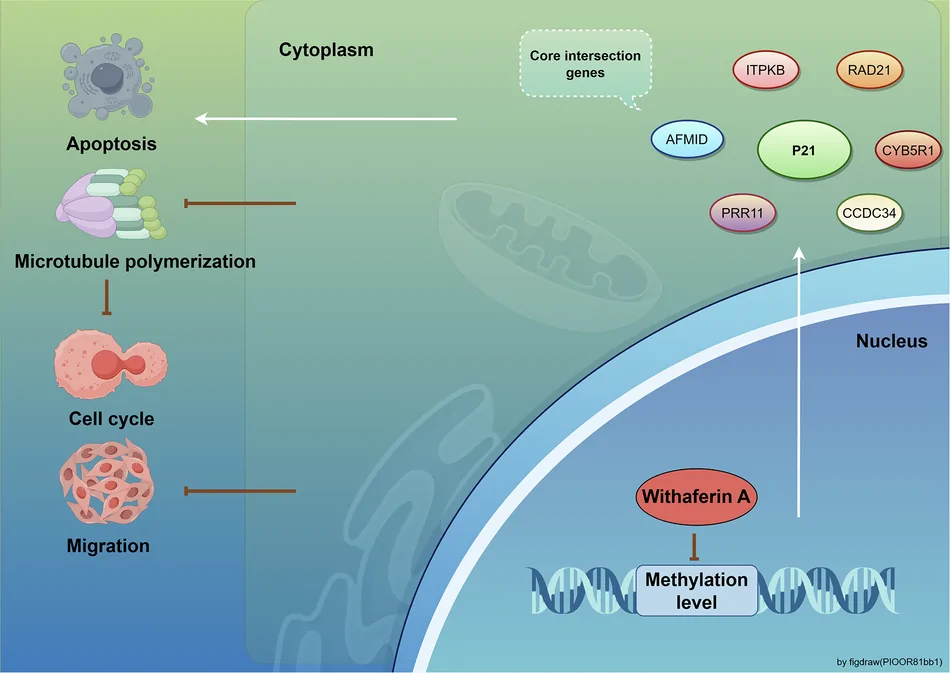

## Introduction

T-cell acute lymphoblastic leukemia (T-ALL) is an aggressive hematologic malignancy that originates from the accumulation of genetic lesions during T-cell development in the thymus, leading to differentiation arrest and uncontrolled proliferation of immature progenitor cells [1]. In recent years, the implementation of risk-based stratification and intensive multi-agent chemotherapy regimens has significantly improved overall survival in children and young patients [2]. However, the current treatment strategies are associated with high toxicity, resulting in an overall survival rate of less than 50% in adult patients [3]. Therefore, the development of novel therapeutic approaches is urgently needed.

**Fig. 1:**
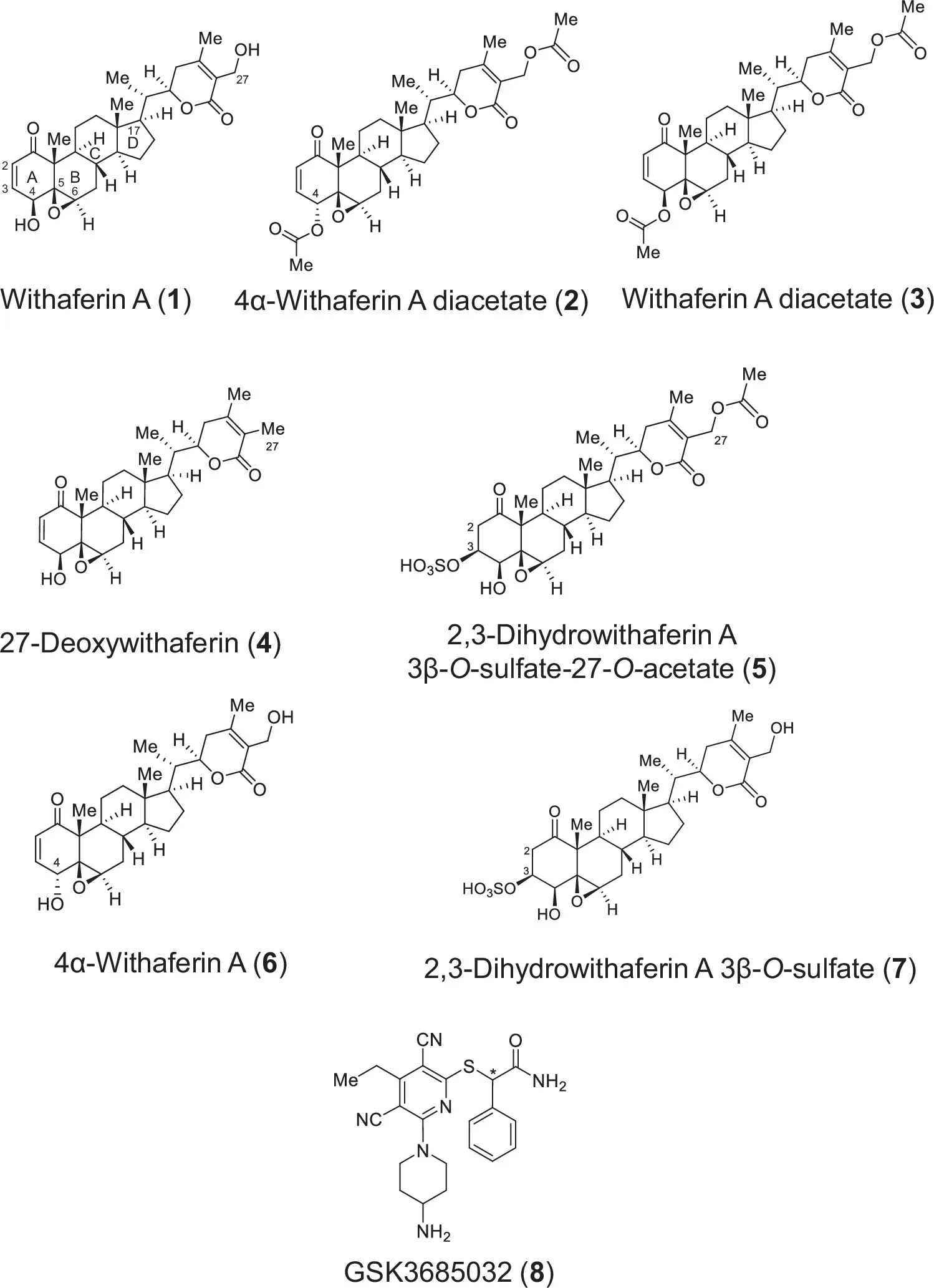
Chemical structures of withaferin A (1) and its six analogs, compounds 2-7. Compound **8** is the DNMT1 positive inhibitor GSK3685032 (racemic mixture), used as a positive control.

*Withania somnifera* is a medicinal herb from the Solanaceae family [4, 5], which is highly regarded for its bioactivity, including its anticancer effects [6, 7]. Withanolides, the major constituents and pharmacologically active steroidal lactones of *W. somnifera*, share a common ergostane skeleton (a C₂₈ steroid) [8]. They exert remarkable antitumor, anti-inflammatory, immunomodulatory, antioxidant, and cognitive-enhancing activities, which are often related to the lactone ring of withanolides and their specific hydroxylation patterns [9]. Withaferin A (compound **1**, for the structure see Fig. 1), a well-known steroidal lactone, is one of the most prominent withanolides extracted from *W. somnifera* [10, 11]. In addition to its roles in anticoagulation, antiplatelet aggregation, antiparasitic activity, anti-adipogenesis, and anti-pigmentation, withaferin A has demonstrated potent and broad-spectrum anticancer effects [12–16]. Both in vitro and in vivo studies have reported that withaferin A (**1**) promotes cancer cell apoptosis, inhibits inflammation and fibrosis, enhances chemosensitivity of cancer cells, and exhibits a favorable safety profile, with good tolerability in mice [17–22]. In recent years, the epigenetic and DNA methylation-related effects of withaferin A have attracted increasing attention in the investigation of its pharmacological mechanisms. Notably, co-treatment with phenethyl isothiocyanate or sulforaphane has been shown to modulate multiple epigenetic pathways [23, 24]. Additionally, withaferin A has been reported to regulate the methylation status of the promoters of *CDKN1A (p21)* and *CCND1* [25]. However, the underlying mechanisms by which withaferin A modulates DNA methylation remain largely unclear and warrant further investigation.

One of the most prominent epigenetic features of cancer cells is aberrant DNA methylation, characterized by global hypomethylation and hypermethylation of CpG islands in tumor suppressor genes and developmental regulators [26, 27]. DNA methylation is primarily catalyzed by DNMT1, DNMT3A, and DNMT3B [27, 28], among which DNMT1 is responsible for maintaining existing methylation patterns, whereas DNMT3A and DNMT3B are involved in establishing de novo methylation at CpG dinucleotides [29, 30]. Notably, DNA methylation is a reversible process; the level of DNA methylation thus represents a potential therapeutic target in cancer treatment.

Therefore, this study aimed to comprehensively re-explore the pharmacological mechanisms of withaferin A (**1**) in T-cell acute lymphoblastic leukemia. Specifically, we seek to elucidate the effects of withaferin A on DNA methylation, identify its key target proteins, and uncover the critical genes that influence its pharmacological activity.

## Results

### Withaferin A (1) and its derivatives inhibit cancer cell growth

**Fig. 2:**
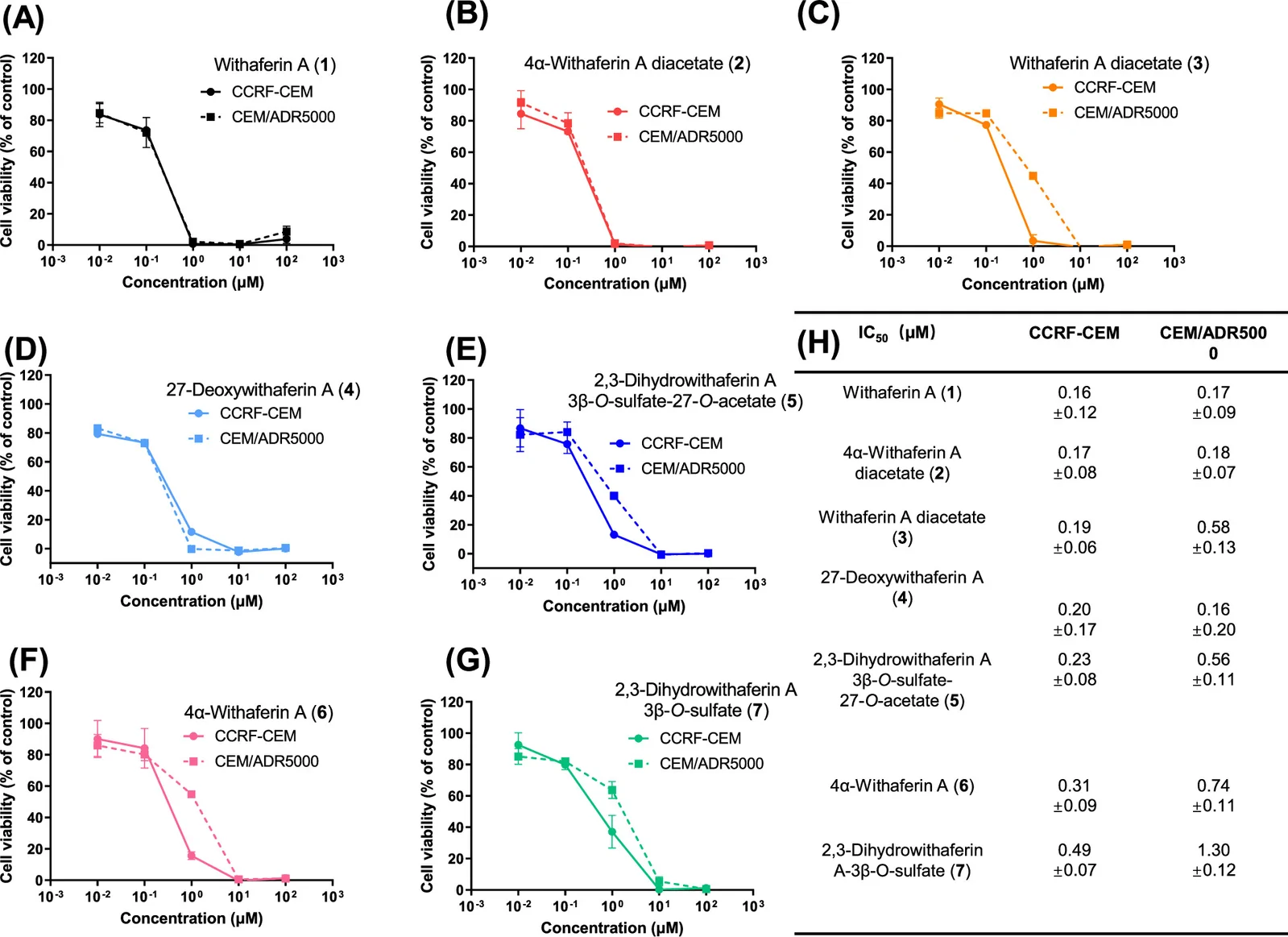
Cytotoxicity of withaferin A (1) and its six analogs in leukemia cells. **A**–**G** Resazurin assays were performed to evaluate the cytotoxicity of withaferin A (**1**), 4α-withaferin A diacetate (**2**), withaferin A diacetate (**3**), 27-deoxywithaferin A (**4**), 2,3-dihydrowithaferin A 3β-*O*-sulfate-27-*O*-acetate (**5**), 4α-withaferin A (**6**), and 2,3-dihydrowithaferin A 3β-*O*-sulfate (**7**) in CCRF-CEM and CEM-ADR5000 cells. **H** IC_50_ values of withaferin A (**1**) and its six analogs, compounds **2-7**, in CCRF-CEM and CEM/ADR5000 cells (mean ± SD, *n* = 3).

We investigated the impact of structural modifications of withaferin A (**1**) on the cytotoxicity using a resazurin reduction assay in CCRF-CEM and CEM/ADR5000 leukemia cells. Among all tested compounds, withaferin A (**1**) exhibited the strongest cytotoxicity against CCRF-CEM cells, with an IC₅₀ value of 0.16 ± 0.12 µM, followed by 4α-withaferin A diacetate (**2**, IC₅₀ = 0.17 ± 0.08 µM) and withaferin A diacetate (**3**, IC₅₀ = 0.19 ± 0.06 µM) as shown in Fig. 2A–C. The reduced cytotoxicity of compound **3** compared with that of withaferin A (**1**) can be attributed to the *O*-acetylation of the free hydroxy groups at C-4 and C-27, highlighting the importance of these underivatized hydroxy groups for cytotoxic activity. Compound **2** largely restored the activity lost in compound **3**, indicating that the α-configuration at C-4 nearly compensated for the effect of double *O*-acetylation. Epimerization at C-4 alone (compound **6**), by contrast, significantly reduced the cytotoxicity, increasing the IC₅₀ from 0.16 µM to 0.31 µM. The free C-27 hydroxy group also contributes to cytotoxic activity, as its removal in compound **4** reduced the activity (IC₅₀ = 0.20 ± 0.17 µM). Addition of sulfuric acid to the C-2/C-3 double bond in the A ring further decreased cytotoxicity in compound **7** (IC₅₀ = 0.49 ± 0.07 µM), whereas subsequent 27-*O*-acetylation partially restored activity in compound **5** (IC₅₀ = 0.23 ± 0.08 µM).

In CEM/ADR5000 cells, 27-deoxywithaferin A (**4**) exhibited the strongest cytotoxicity (IC₅₀ = 0.16 ± 0.20 µM), closely followed by withaferin A (**1**) (IC₅₀ = 0.17 ± 0.09 µM; Fig. 2D–F). 2,3-Dihydrowithaferin A 3β-*O*-sulfate (**7**), on the contrary, showed the weakest activity in both CCRF-CEM and CEM/ADR5000 cells (Fig. 2G). Overall, withaferin A (**1**) and 4α-withaferin A diacetate (**2**) were the most active compounds (IC₅₀ < 0.2 µM) in both investigated leukemia cell lines (Fig. 2H). As withaferin A (**1**) consistently displayed lower IC₅₀ values than compound **2**, it was identified as the most potent compound in this study.

### Withaferin A (**1**) inhibits the growth of various cancer cell lines

**Fig. 3:**
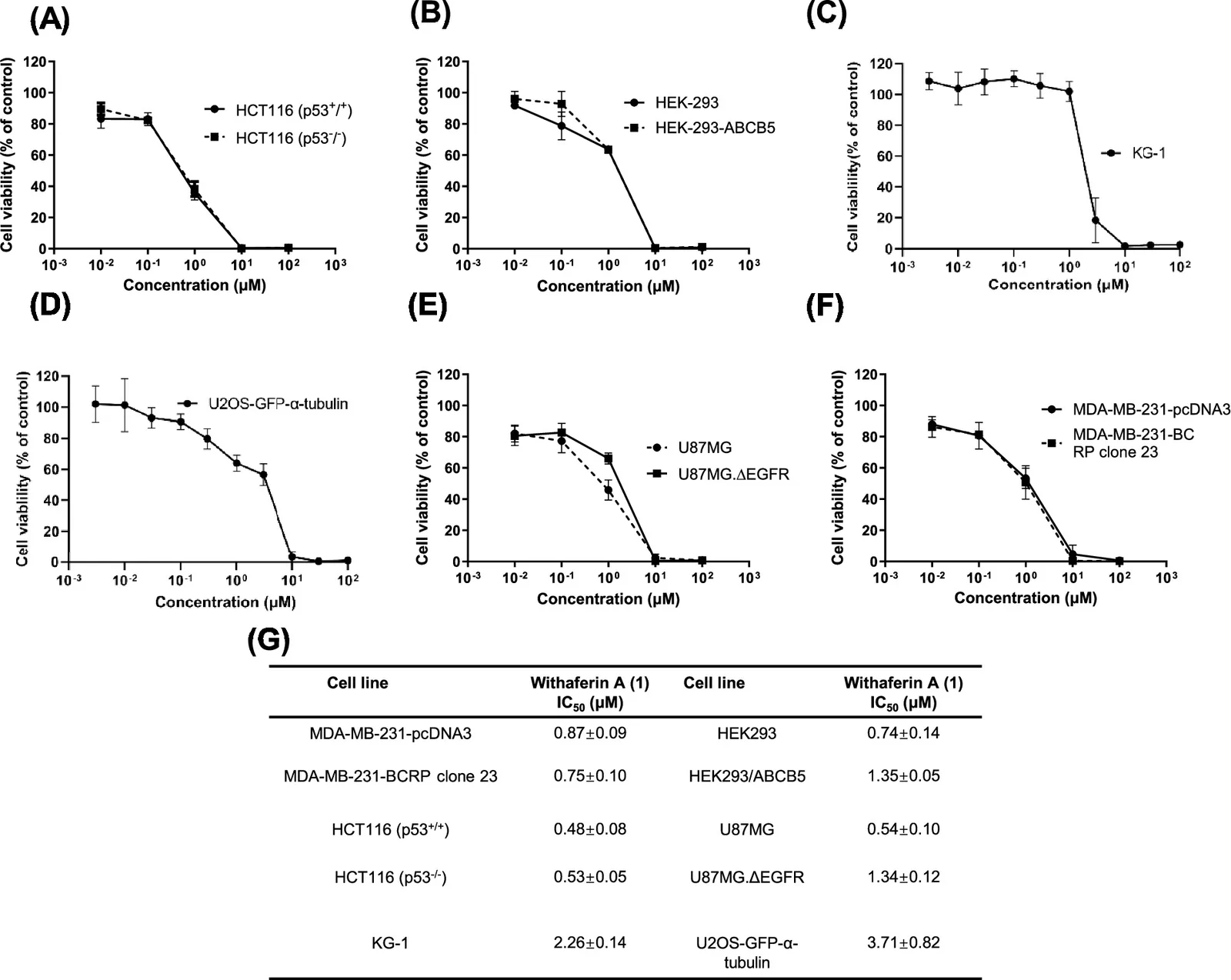
Cytotoxicity of withaferin A (1) in various types of cancer cells. **A**–**F** Resazurin assays were performed to assess the cytotoxicity of withaferin A in breast cancer, colon cancer, glioblastoma multiforme, HEK293T, and osteosarcoma cells. **G** IC_50_ values of withaferin A (**1**) in different cancer cell types (mean ± SD, *n* = 3).

The cytotoxicity of withaferin A (**1**) was evaluated across multiple cancer cell lines using the resazurin reduction assay. Among these, withaferin A (**1**) exhibited the strongest cytotoxicity against CCRF-CEM and CEM/ADR5000, with IC₅₀ values of 0.16 ± 0.12 μM and 0.17 ± 0.09 μM, respectively (Fig. 2A). In addition, the IC₅₀ values were determined for U2OS cells stably expressing α-tubulin-GFP (used in migration assays) and KG-1 cells (used in luciferase reporter assays), which were 3.71 ± 0.83 μM and 2.26 ± 0.14 μM, respectively. In addition, the IC_50_ values of withaferin A for HCT116 (p53^+/+^) and HCT116 (p53^–/–^) cells were 0.48 ± 0.08 μM and 0.53 ± 0.05 μM, respectively. The weakest cytotoxicity, however, was observed in HEK293 and HEK293/ABCB5 cells, with IC₅₀ values of 0.74 ± 0.14 μM and 1.35 ± 0.05 μM, respectively (Fig. 3A–D). The cytotoxicity results for the other cell lines are shown in Fig. 3E and 3F, and IC_50_ values are summarized in the table (Fig. 3G). Notably, the CCRF-CEM cell line, derived from a T-ALL patient, was identified as the most sensitive cell line towards withaferin A (**1**) among all tested cancer cell lines (Fig. 3G). The LIVE/DEAD flow cytometric viability assay also demonstrated significant differences in the proportions of live and dead cells in all four cell lines. In KG-1 and CCRF-CEM cells, treatment with 1 × IC₅₀ and 2 × IC₅₀ of withaferin A reduced the percentage of live cells by more than 50%, accompanied by a corresponding increase in the proportion of dead cells (*p* < 0.05). In U2OS cells, treatment with 1 × IC₅₀ (3.71 μM) and 2 × IC₅₀ (7.42 μM) of withaferin A (**1**) resulted in a reduction of the live-cell population by more than 15%, with a corresponding increase in dead cells (*p* < 0.05). In HCT116 (p53^+/+^) cells, treatment with 2 × IC₅₀ (0.96 μM) of withaferin A induced only a modest reduction of approximately 8% in the live-cell population, accompanied by a corresponding increase in dead cells (*p* < 0.05) as shown in Fig. S2A. To identify sub-toxic concentrations, the viability of HCT116 (p53^+/+^) cells was first evaluated following treatment with low concentrations of withaferin A (**1**). Concentrations maintaining > 90% cell viability (0.01 and 0.03 μM) were selected as sub-toxic concentrations for the colony formation assay. Figure S2B demonstrated that withaferin A (**1**) at 0.03 μM significantly inhibited the clonogenic capacity of HCT116 cells (*p* < 0.05).

### Identification of Key Target Genes: Exploring the Potential Mechanism of Withaferin A (1) in T-ALL

**Fig. 4:**
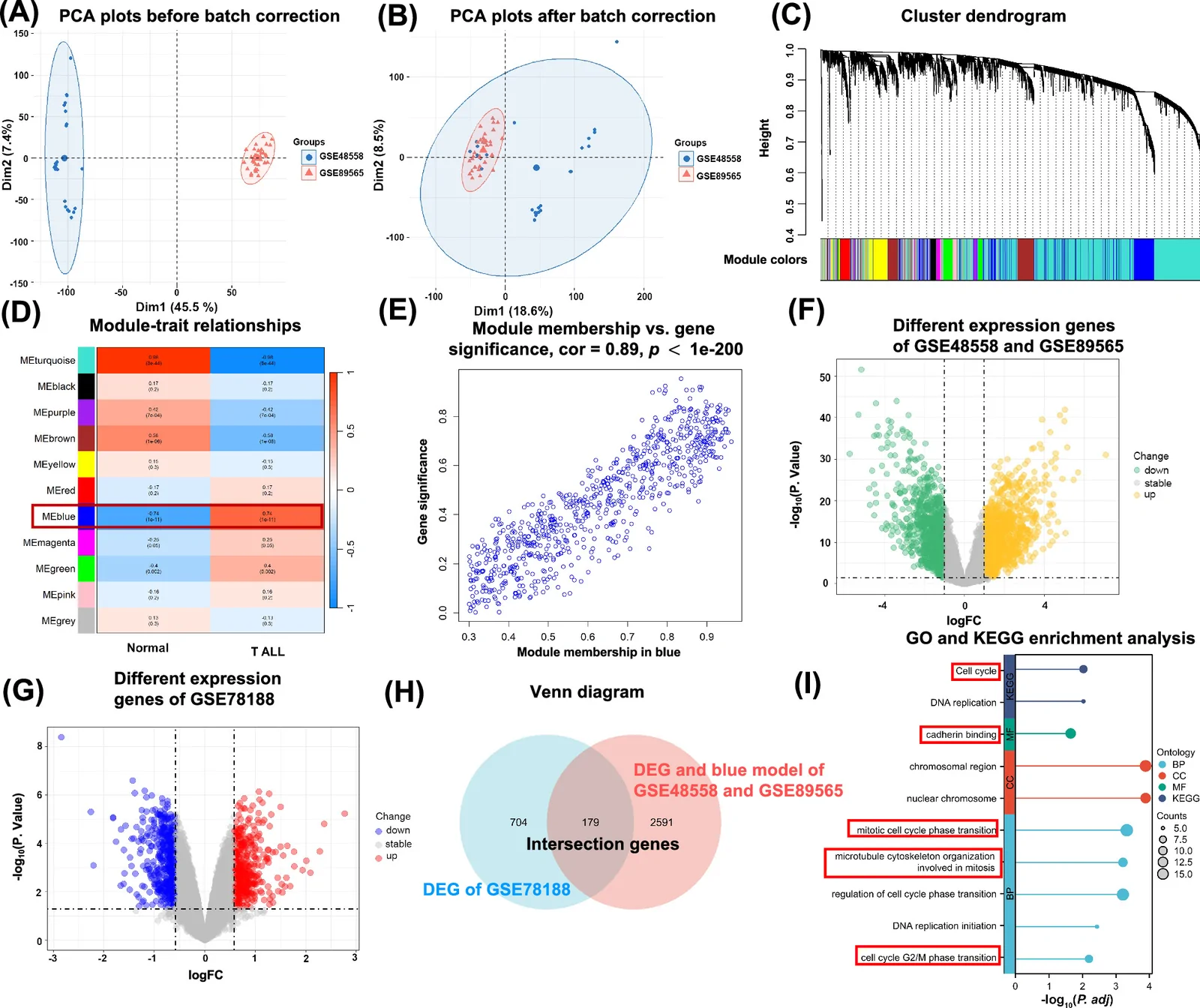
Identification of key genes associated with withaferin A-mediated leukemia inhibition and their biological functions. **A**, **B** PCA plots before and after batch correction. **C** Gene clustering dendrogram based on network interconnectivity. **D** Heatmap showing the correlations between gene modules and clinical traits. **E** Correlation between module membership and gene significance in the blue module. **F** Differentially expressed genes (DEGs) in GSE48558 and GSE89565. **G** DEGs in GSE78188. **H** Key genes involved in the pathogenic inhibition of leukemia by withaferin A. **I** GO and KEGG enrichment analysis of the key genes.

To identify potential target genes of withaferin A-treated T-cell acute lymphoblastic leukemia (T-ALL), two microarray datasets were integrated and analyzed, GSE48558 and GSE89565, which together include 17 normal T-cell samples and 43 T-ALL samples. PCA revealed a pronounced batch effect between the two datasets (Fig. 4A). After batch correction using the removeBatchEffect function from the limma package, the systematic variation among samples was markedly reduced, and the corrected PCA showed clear separation between normal T cells and T-ALL samples along the principal components (Fig. 4B). A weighted gene co-expression network analysis (WGCNA) was subsequently performed based on the expression matrix. The top 5,000 genes with the highest median absolute deviation (MAD) values were used to construct the network, resulting in 11 distinct modules (Fig. 4C). Correlation heatmap analysis indicated that the blue module exhibited the strongest positive correlation with the clinical trait T-ALL (Fig. 4D), suggesting that this module may represent a set of genes closely associated with disease progression. Furthermore, the gene significance (GS) *vs*. module membership (MM) scatter plot revealed a positive correlation within the blue module, as shown in Fig. 4E. This further indicated a strong association between the genes within the blue module and the clinical trait T-ALL. Differential expression analysis between normal T cells and T-ALL samples in GSE48558 and GSE89565 revealed numerous significantly altered genes (Fig. 4F). In parallel, the dataset GSE78188, which consists of CUTLL cells - a novel T-ALL cell line - treated with either withaferin A (**1**) or DMSO, was analyzed. Differentially expressed genes (DEGs) induced by withaferin A treatment were identified and blotted in Fig. 4G. The DEGs from GSE48558 and GSE89565 were combined with the genes in the blue module associated with T-ALL to generate a list of candidate T-ALL pathogenic genes. These were then intersected with the DEGs from GSE78188, resulting in the identification of 179 overlapping genes as presented in Fig. 4H. These genes are considered potential key targets through which withaferin A may exert its inhibitory effects on T-ALL pathogenesis. The GO and KEGG enrichment analyses were performed on the 179 key genes using the R package clusterProfiler. Interestingly, the results revealed significant enrichment in biological processes and pathways, including cell cycle, cadherin binding, mitotic cell cycle phase transition, and microtubule cytoskeleton organization involved in mitosis, as listed in Fig. 4I.

### In single-cell analysis, blasts represent the cell cluster most strongly influenced by key genes

**Fig. 5:**
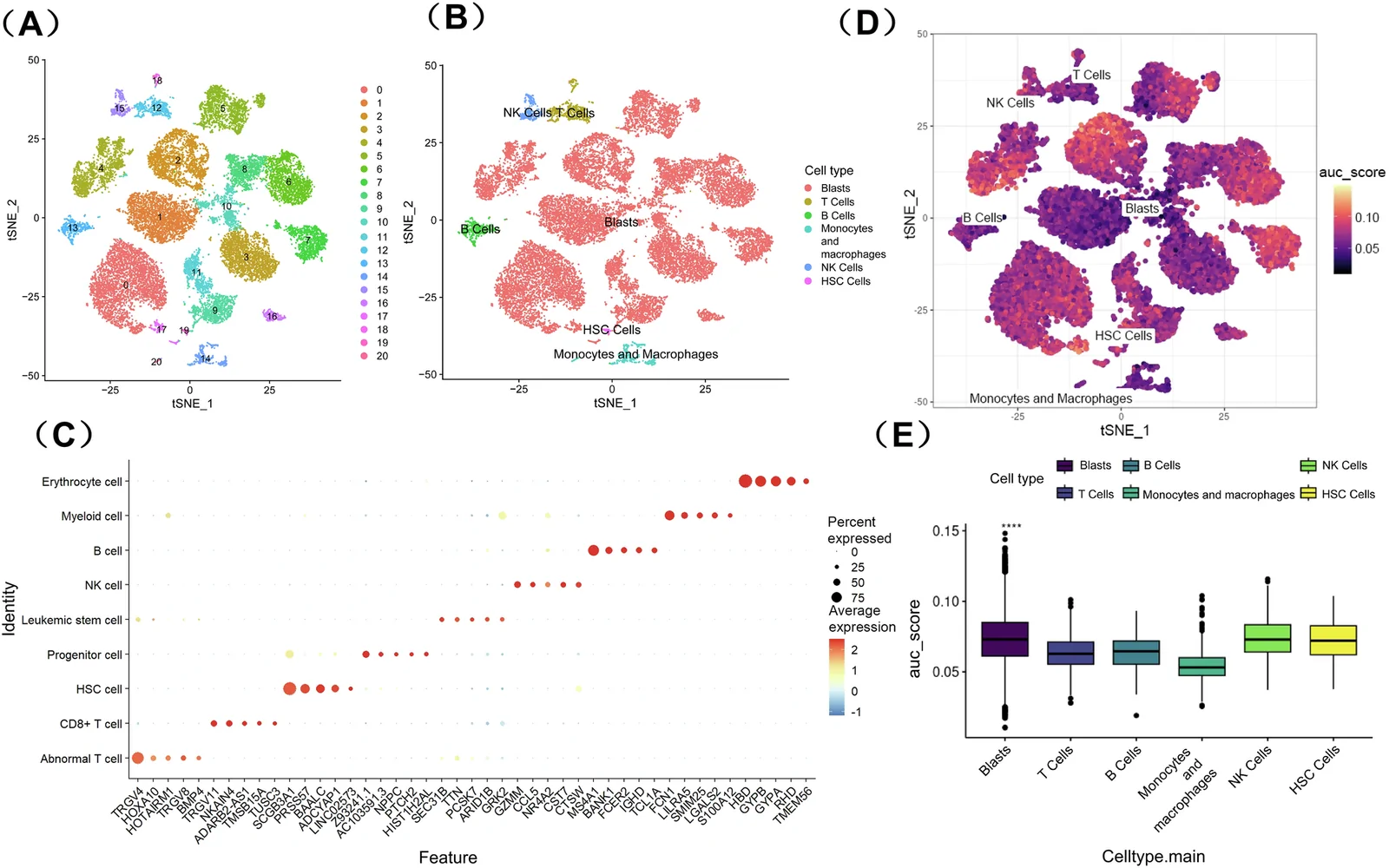
In single-cell analysis, blasts represent the cell cluster most strongly influenced by key genes. **A** t-SNE plot of all cell clusters. **B** Cell type annotation. **C** The top five marker genes for each cell type. **D**, **E** AUCell-based scoring of these cell clusters.

To determine which cell types the 179 core gene set primarily functions in T-ALL patients, ten T-ALL samples from the GSE227122 dataset were reanalyzed. Then, based on the clustering tree, cells were grouped into 20 clusters at a resolution of 0.5 (Fig. 5A). These clusters were further annotated into six major cell types using marker genes from the SingleR algorithm and the CellMarker database (Fig. 5B). Marker genes for each of the six cell types were identified using the FindAllMarkers() function (Fig. 5C). To assess the activity of key target genes regulated by withaferin A (**1**), AUCell was applied to score each cell based on a curated list of 179 target genes. The results showed that blast cells exhibited the highest AUCell scores among all cell types (Fig. 5D, E), suggesting that withaferin A-associated target genes exert their most prominent functional effects in leukemic blasts from T-ALL patients.

### Microarray validation and IPA functional analysis

**Fig. 6:**
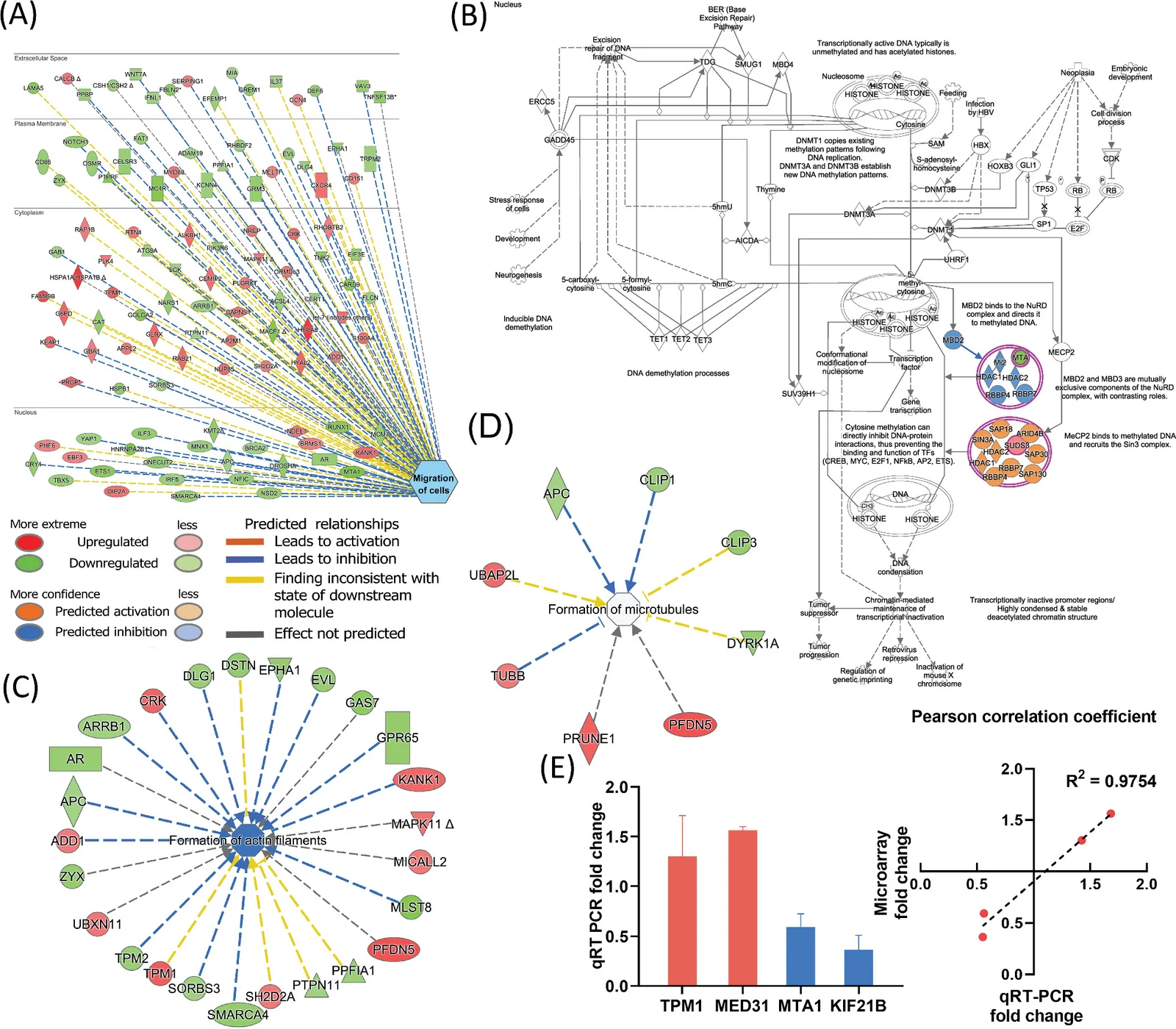
IPA analysis of gene regulatory networks affected by withaferin A (1) and microarray validation. **A**–**D** IPA analysis of gene regulatory networks and biological functions affected in CCRF-CEM cells upon treatment with withaferin A (**1**). **E** qRT-PCR validation of selected genes from the microarray, including Pearson correlation analysis of microarray and qRT-PCR data.

To investigate the biological functions and pathways affected by withaferin A (**1**) in T-ALL cells, gene expression microarray analysis was performed on CCRF-CEM cells treated with withaferin A, followed by Ingenuity Pathway Analysis (IPA) of the resulting data. CCRF-CEM cells were divided into two groups and treated with either DMSO (control) or withaferin A at its IC₅₀ concentration for 24 h. Total RNA was extracted and subjected to microarray analysis. Moreover, to explore the potential biological mechanisms underlying withaferin A activity, IPA software was used on the differentially expressed genes (DEGs) identified from the microarray data. A total of 352 genes were significantly upregulated, and 417 genes were downregulated. IPA predicted that, compared to the DMSO group, withaferin A treatment significantly inhibited the cellular migration function (Fig. 6A) and strongly modulated pathways related to DNA transcription. *HDAC1* and *HDAC2* were predicted as key regulatory factors in these pathways. Moreover, *DNMT1, DNMT3A*, and *DNMT3B* were centrally located within the transcriptional regulation network, suggesting that these DNA methyltransferases may play critical roles in the epigenetic modulation mediated by withaferin A (Fig. 6B). Figure 6C, D further show that withaferin A inhibits the formation of actin filaments and microtubules. Specifically, in the microtubule formation pathway, *APC, CLP1, CLP3*, and *DYRK1A* were predicted to be downregulated. Additionally, *UBAP2L, TUBB, PRUNE1*, and *PFDN5* also exhibited predicted negative regulation, suggesting that withaferin A may suppress microtubule dynamics through the coordinated inhibition of multiple targets. The downregulation of *KIF21B* was predicted to suppress acute lymphoblastic leukemia. In addition, the gene network containing *MED31* and *MTA1* was associated with the cohesin chromatin regulation pathway and DNA methylation and transcriptional repression signaling (Supplementary Figs. 3 and 4). To validate the microarray results, *TPM1, MED31, MTA1*, and *KIF21B*, which were among the top ten most significantly up- or downregulated genes within these networks, were selected for RT-qPCR analysis. The correlation between fold changes from microarray and qPCR was calculated, showing a high concordance, with an R² value of 0.99 (Fig. 6E).

### Withaferin A (**1**) reduces DNA methylation levels in KG-1 cells and microarray dataset

**Fig. 7:**
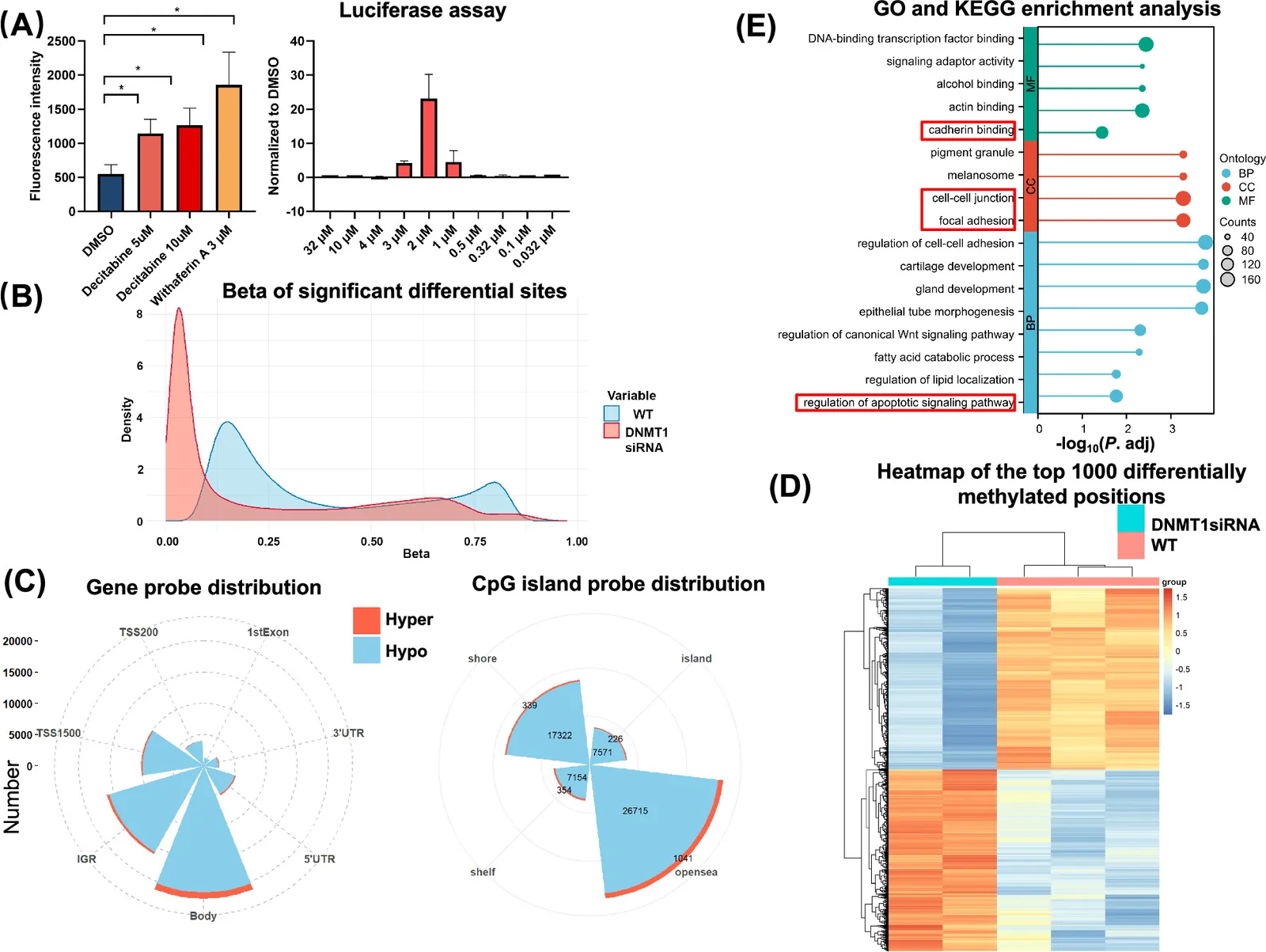
Withaferin A (1) reduces DNA methylation levels. **A** Luciferase reporter assay detecting the effect of withaferin A on gene transcriptional activity (mean ± SD, *n *= 3, *p* < 0.05). **B** Density plots illustrating global methylation levels in wild-type *vs*. DNMT1 knockdown groups. **C** Radar plot showing the distribution of differentially methylated probes across genomic regions. **D** Heatmap of the top 1000 differentially methylated positions. **E** GO and KEGG enrichment analysis of the top 5000 genes annotated by differentially methylated positions.

To investigate the effect of withaferin A (**1**) on DNA methylation levels, a luciferase reporter assay was performed in KG-1 cells. The results showed that the positive control, decitabine (5 and 10 μM), as well as withaferin A (**1**) at 3 μM, significantly enhanced reporter activity compared with the DMSO group (*p* < 0.05), indicating that **1** suppresses cytomegalovirus promoter methylation of the target construct. We further explored the most effective concentration of withaferin A (**1**) for modulating DNA methylation levels. The results demonstrated that 2 μM of withaferin A induced the greatest change in DNA methylation levels compared with the DMSO control (Fig. 7A). The maintenance of DNA methylation at tumor suppressor genes by DNMT1 has been shown to contribute to tumorigenesis in various types of cancer, and withaferin A has been reported to suppress DNMT1 protein expression [31–34]. To further investigate the inhibitory effect of withaferin A on DNMT1, a DNMT1 activity inhibition assay was performed. The results showed that withaferin A (**1**) at 5 and 10 μM did not inhibit DNMT1 activity (Supplementary Fig. S1). In addition, the DNA methylation microarray dataset GSE104359, which contains three wild-type and two DNMT1-knockdown samples, was reanalyzed. The results demonstrated that, compared with the wild type, the DNMT1 knockdown group exhibited a marked global decrease in DNA methylation levels (Fig. 7B). Genome-wide distribution analysis revealed that the differentially methylated CpG sites (|Δβ | ≥ 0.2, FDR (adj.P.Val) < 0.05) were predominantly located in open sea regions far from CpG islands and in shore regions at the CpG island periphery. Regarding gene structure, most of these sites were located within the body region of the gene (exons/introns) and intergenic regions (IGRs) (Fig. 7C). Furthermore, the top 1000 CpG sites with the largest positive Δβ values and the top 1000 with the largest negative Δβ values were selected, Z-score normalization was performed using the scale() function, and a heatmap was generated to visualize the relative methylation levels across samples (Fig. 7D). The top 5000 CpG sites ranked by |Δβ| were selected, mapped to annotated genes, and submitted to GO enrichment analysis. The results indicated that DNMT1 knockdown primarily affected biological processes such as cadherin binding, cell-cell junction, focal adhesion, and regulation of the apoptotic process (Fig. 7E).

### Withaferin A (**1**) promotes apoptosis as well as cell-cycle arrest, and inhibits cell migration

**Fig. 8:**
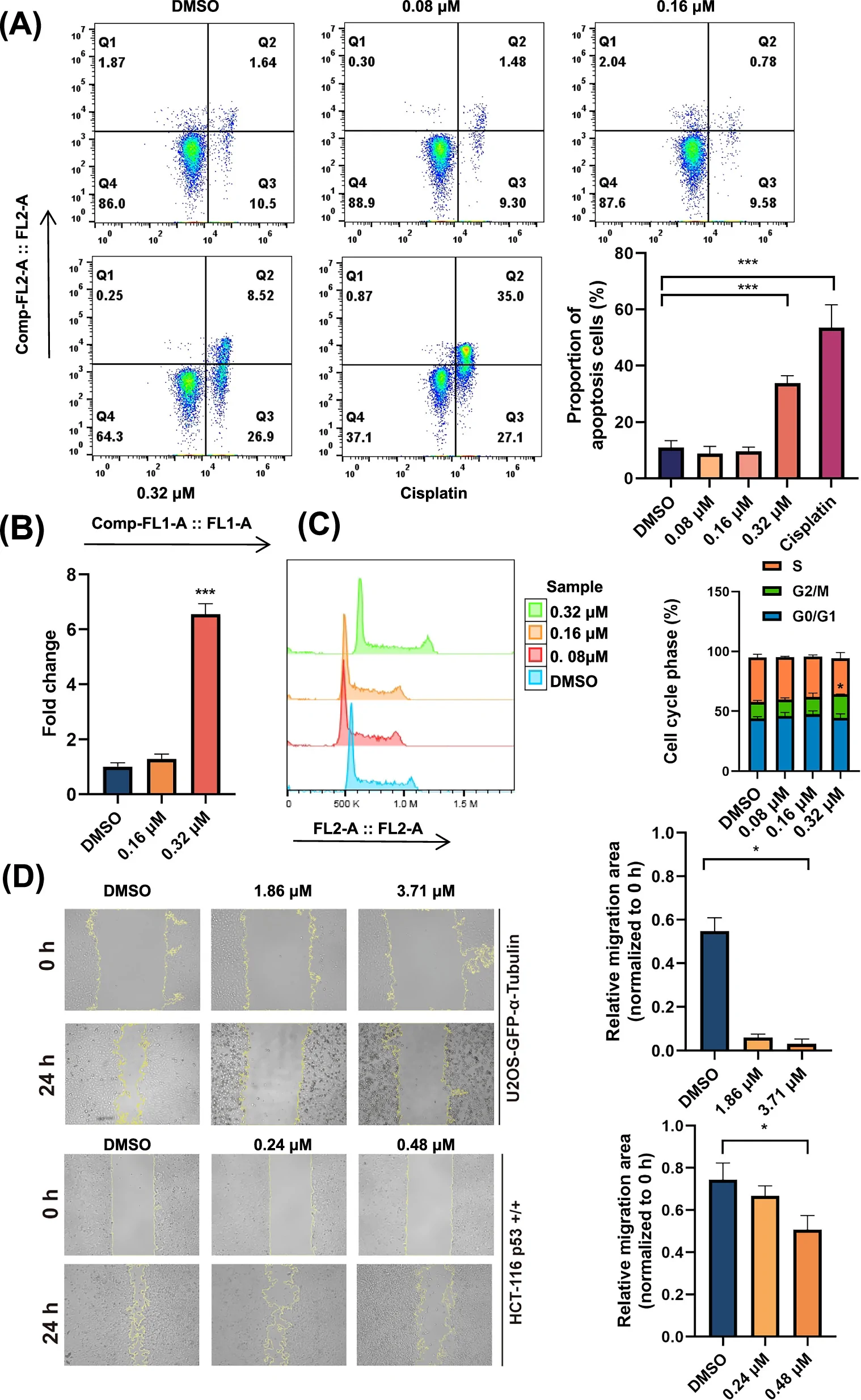
Withaferin A (1) promotes apoptosis as well as cell-cycle arrest, and inhibits cell migration. **A** Flow cytometry analysis of changes in apoptosis levels. **B** qPCR analysis of changes in *CDKN1A* (p21) RNA expression levels. **C** Flow cytometry analysis of the cell cycle. **D** Wound-healing assay detecting the effect of withaferin A on the cell migration rate. Each experiment was repeated with three independent replicates. Mean ± SD, *p < 0.05; **p < 0.01; ***p < 0.001.

To validate the biological functions of withaferin A (**1**) in T-ALL cells and to further explore its underlying mechanisms, a series of functional assays were conducted. Annexin V/PI staining demonstrated that CCRF-CEM cells, compared with the DMSO control group, treated with withaferin A (0.32 μM, 2 × IC₅₀, 48 h) or cisplatin as the positive control, significantly increased the proportion of apoptotic cells, including both early and late apoptosis (*p* < 0.05) (Fig. 8A). qRT-PCR analysis revealed that expression of *P21* was significantly upregulated in the CCRF-CEM treated with 2 × IC₅₀ (0.32 μM) of withaferin A compared with the DMSO control (*p* < 0.05) as is displayed in Fig. 8B. Western blot analysis revealed significantly increased p53 protein expression following treatment with 2 × IC₅₀ (0.32 μM) of withaferin A (**1**) and significantly increased p21 protein expression following treatment with 1 × IC₅₀ (0.16 μM) and 2 × IC₅₀ (0.32 μM) (*p* < 0.05, Fig. S2C). Flow cytometry further confirmed that withaferin A (2 × IC₅₀, 0.32 μM) induced significant G2/M phase arrest, as shown in Fig. 8C, suggesting that withaferin A mediates cell-cycle arrest in CCRF-CEM cells via upregulation of *P21* expression with *p* < 0.05. Wound-healing assays demonstrated that in U2OS-GFP-α-tubulin and HCT116 p53^+/+^ cells, treatment with withaferin A (3.71 μM, 0.48 μM) significantly reduced cell migration ability compared with the DMSO group, with *p* < 0.05 (Fig. 8D).

### Withaferin A (**1**) distorts the microtubule network and regulates the expression of core intersection genes

**Fig. 9:**
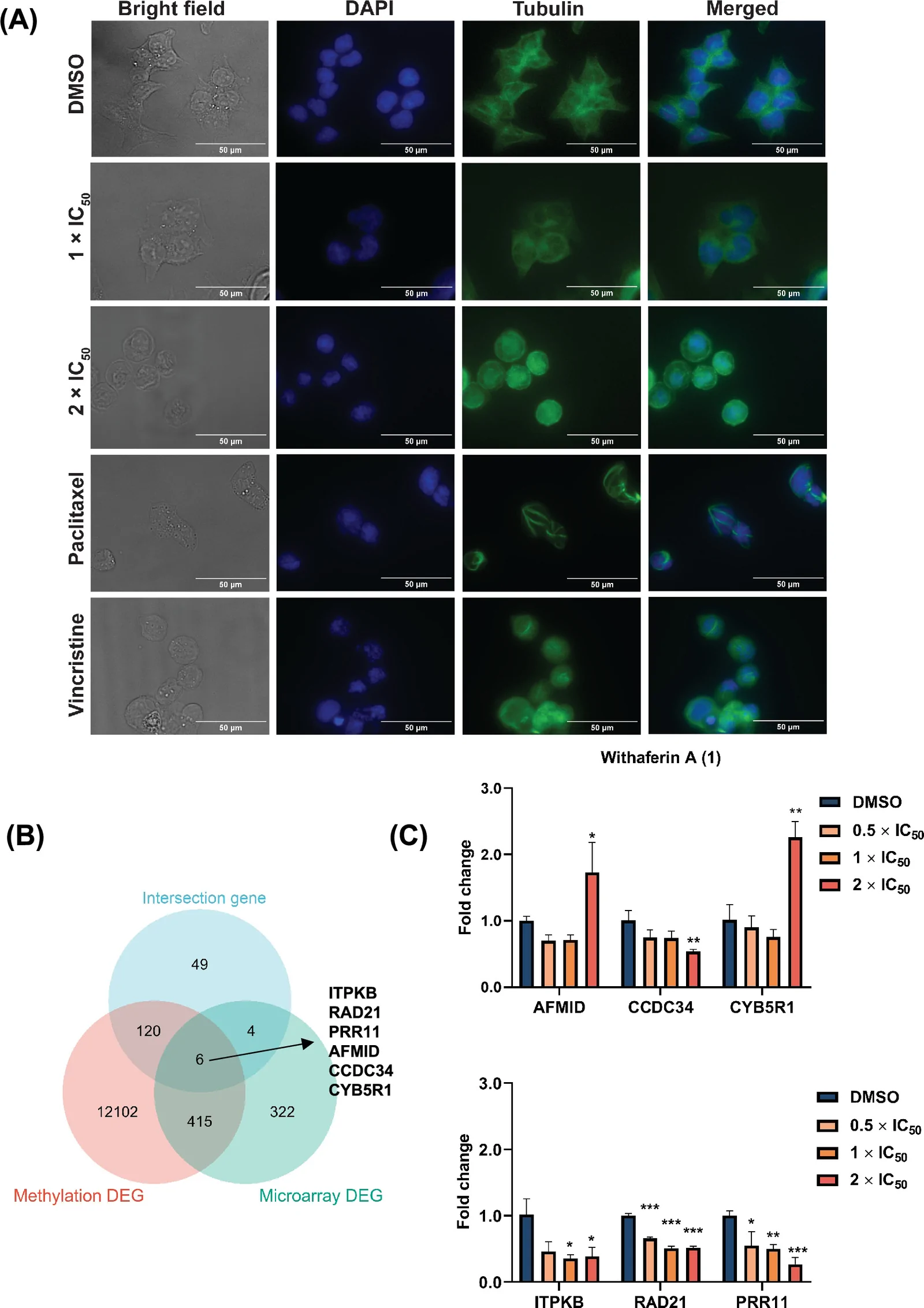
Withaferin A (1) distorts the microtubule network and regulates the expression of core intersection genes. **A** Immunofluorescence imaging of microtubule morphology. **B** Venn diagram showing six intersecting genes among key genes, genes annotated by differentially methylated sites, and microarray DEGs. **C** qPCR validation of the six overlapping genes after treatment with withaferin A. Each experiment was repeated with three independent replicates (mean ± SD). *p < 0.05; **p < 0.01; ***p < 0.001.

Immunofluorescence staining was used to determine whether withaferin A (**1**) induces mitotic arrest and interferes with microtubule polymerization. In the DMSO control group, microtubules extended continuously throughout the cytoplasm and formed an intact network, indicating proper polymerization. In the paclitaxel-treated positive control, the microtubule network appeared rigid and resistant to depolymerization. Vincristine was selected as the positive control for suppressing microtubule polymerization because it potently inhibits tubulin addition at microtubule ends [35]. Both vincristine and withaferin A (2 × IC_50_) treatment resulted in reduced microtubule extension at the cell periphery and increased tubulin accumulation around the nucleus. These findings suggest that withaferin A, similar to vincristine, disrupts the microtubule network and suppresses microtubule polymerization (Fig. 9A). Furthermore, by intersecting the previously identified 179 overlapping genes with differentially methylated genes from GSE104359 and differentially expressed genes from the microarray dataset, six core genes were identified, as shown in Fig. 9B. qPCR validation revealed significant downregulation of ITPKB, RAD21, CCDC34 and PRR11, and significant upregulation of AFMID, and CYB5R1 (Fig. 9C).

### Antitumor activity of withaferin A (**1**) in zebrafish models

**Fig. 10:**
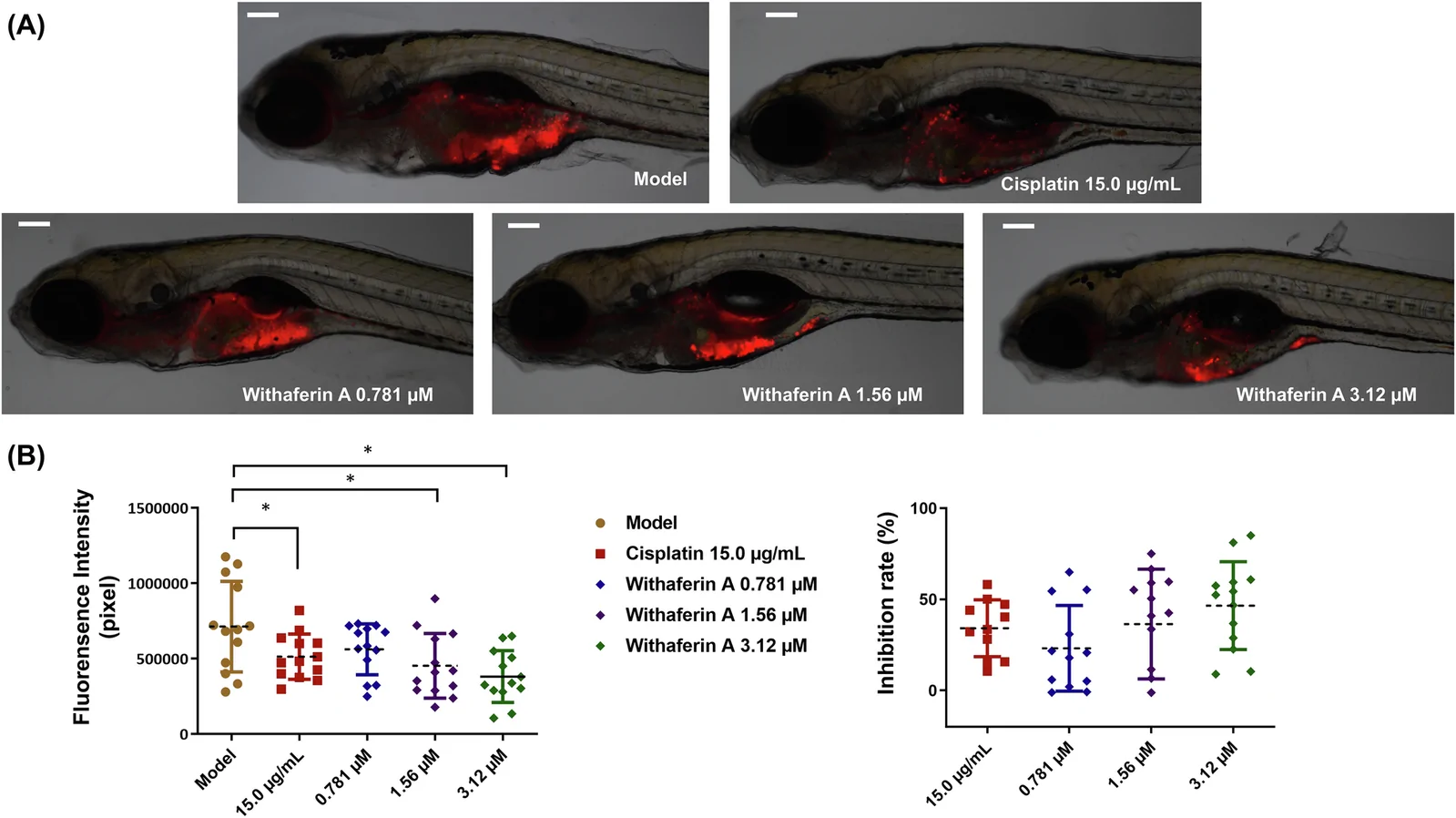
Antitumor activity of withaferin A (1) in zebrafish models. **A** Flourensence images of CCRF-CEM xenografts in zebrafish. Red fluorescence indicates the size of the CCRF-CEM tumor mass in each group. **B** Quantification of  fluorescence intensity and corresponding inhibition rates after 48 h of treatment with the control, cisplatin, and withaferin A at two concentrations (mean ± SD). *p < 0.05; **p < 0.01; ***p < 0.001.

A CCRF-CEM xenograft zebrafish model was established to evaluate the antitumor activity of withaferin A (**1**). Three different concentrations of withaferin A (**1**) were tested in this model. The growth of xenografted tumor cells was assessed based on cellular fluorescence intensity. After 48 h of treatment, compared with the DMSO control, cisplatin (15 μg/mL) and withaferin A (**1**) at 1.56 and 3.12 μM significantly inhibited the growth of CCRF-CEM tumors, with inhibition rates of 34.12%, 36.46%, and 46.55%, respectively (Fig. 10A, B).

## Discussion

As part of our ongoing efforts to re-examine classical natural products, we investigated the structure–activity relationships and cytotoxic profile of withaferin A (**1**), a naturally occurring C_28_-steroidal lactone. Its characteristic electrophilic enone and epoxide moieties and the free allylic hydroxy groups at C-4 and C-27 were systematically modified to assess their contribution to the cytotoxic activity. Overall, the parent compound retained the highest activity among the tested derivatives, suggesting that the natural combination of functional groups in withaferin A represents an optimal structural arrangement for cytotoxicity. Given its previously reported activity against cancers of the reproductive, digestive, endocrine, and nervous systems [10], we further evaluated withaferin A (**1**) across 12 cancer cell lines. Among them, the T-ALL-derived CCRF-CEM cells were the most sensitive.

The increased sensitivity of leukemia cells to withaferin A (**1**) may be attributed to their rapid proliferative nature. Unlike most solid tumors, acute leukemia is characterized by uncontrolled proliferation of immature hematopoietic cells [36]. Such rapid proliferation requires continuous cell-cycle progression and accurate mitosis, rendering leukemia cells more vulnerable to agents that disrupt mitotic progression [37]. Consistent with this notion, our transcriptomic analysis, as well as cell-cycle and immunofluorescence experiments, demonstrated that withaferin A (**1**) induced G2/M arrest and disrupted the microtubule network, indicating that interference with mitotic progression contributes to its enhanced activity against leukemia cells. In addition, epigenetic regulation may provide an additional explanation. Aberrant DNA methylation is a hallmark of leukemia and plays a central role in leukemogenesis [38–40], as demonstrated by the clinical efficacy of DNMT inhibitors such as decitabine [41]. Previous studies have shown that withaferin A (**1**) can modulate the expression and global activity of DNMTs and HDACs [42], Consistently, our luciferase assay suggests that withaferin A regulates cytomegalovirus promoter methylation in leukemia cells.

Furthermore, the cytotoxic effect of withaferin A (**1**) was further confirmed by the LIVE/DEAD assay in all four tested cell lines. Notably, in the LIVE/DEAD assay of HCT116 cells, the decrease in the live-cell population and the increase in the dead-cell population were both below 10% even at 2 × IC₅₀, suggesting that withaferin A (**1**) predominantly inhibited colon cancer cell proliferation rather than inducing extensive cell death within 48 h. This finding is consistent with the withaferin A induced G2/M cell-cycle arrest. Moreover, because LIVE/DEAD staining was performed after 48 h rather than 72 h, some affected cells may not yet have progressed to late apoptosis or necrosis, during which plasma membrane integrity is lost.

Previous studies have employed network pharmacology, computational analysis, proteomics, and metabolomics to investigate the therapeutic mechanisms of withaferin A (**1**) in breast cancer, aging, and Parkinson’s disease; however, such studies remain scarce in leukemia [43–47]. To systematically explore the key gene interaction networks underlying withaferin A treatment in T-ALL, the WGCNA algorithm was applied and identified the blue gene module most strongly associated with T-ALL pathogenesis. By integrating this module with differentially expressed genes from the GSE48558, GSE89565, and GSE78188 datasets, a set of 179 key genes was identified that potentially mediate the inhibitory effects of withaferin A on T-ALL. GO and KEGG enrichment analysis revealed that these genes were predominantly involved in cell cycle regulation, cadherin binding, mitotic cell cycle phase transition, and microtubule cytoskeleton organization during mitosis. Furthermore, single-cell analysis demonstrated that leukemic blasts were markedly more affected by these target genes than other cell types, confirming their critical role in the therapeutic action of withaferin A against T-ALL.

Validation of the predicted functions of these key genes involved in withaferin A–mediated inhibition of T-ALL was performed. The *CDKN1A* gene is transcriptionally activated by the tumor-suppressor transcription factor p53, which arrests cell cycle progression by inhibiting the activity of cyclin-dependent kinases [48, 49]. In this study, qPCR, western blot and cell-cycle analyses indicated that withaferin A (**1**) induces G2/M phase arrest by upregulating p53 and p21 expression. CCRF-CEM cells harbor the hotspot *TP53* missense mutations R175H and R248Q [50]. These two mutations indicate that the translated p53 protein has an impaired ability to control the transcription of target genes and causes the loss of zinc coordination and DNA-binding activity [51, 52]. Therefore, the withaferin A induced upregulation of p21 observed in the present study is unlikely to be mainly mediated by mutant *p53* but is more likely associated with the demethylation of the *p21* promoter. Previous studies have confirmed this mechanism by using CRISPR-dCas9-mediated locus-specific epigenetic editing combined with bisulfite sequencing, demonstrating that restoration of *p21* promoter methylation could significantly reverse the withaferin A induced upregulation of p21 expression [25], which is consistent with our experimental observations. On the other hand, the increased p53 protein expression observed in the present study is more likely to reflect enhanced stability of mutant p53 protein rather than restoration of wild-type p53 function. Previous studies have shown that withaferin A can regulate p53 protein stability by affecting the ubiquitin–proteasome degradation pathway [53]. Microtubules are composed of αβ-tubulin heterodimers that assemble into protofilaments, which laterally associate to form microtubules. This highly dynamic structure continuously elongates and shortens during all phases of the cell cycle by the addition or removal of tubulin dimers at its ends [54, 55]. Immunofluorescence imaging of cancer cells treated with withaferin A further revealed altered β-tubulin morphology, indicating that withaferin A suppresses microtubule polymerization, leading to cell-cycle arrest and ultimately inhibition of mitosis.

Withaferin A (**1**) has been reported to modulate multiple epigenetic pathways when used in combination with phenethyl isothiocyanate or sulforaphane [23, 24, 42]. Evidence also suggests that withaferin A can regulate DNMT1 protein expression; however, the relationship between withaferin A and DNMT1 has so far remained largely at the stage of computational modeling, and its precise molecular mechanism has not yet been clarified [34, 56]. This study provides a comprehensive overview of the alterations in DNA methylation levels induced by withaferin A (**1**) and further explores the downstream regulatory network of DNMT1. We demonstrate that withaferin A reduces DNA methylation levels and, through the downregulation of DNMT1, regulates the expression of multiple target genes, including *P21, ITPKB, RAD21, PRR11, AFMID, CCDC34*, and *CYB5R1*. Withaferin A (**1**) modulated the expression of several genes involved in cell-cycle progression (*PRR11*) [57], cohesin-mediated DNA damage checkpoint activation and repair (*RAD21*) [58, 59], ER-to-mitochondria calcium signaling (ITPKB) [60, 61], tryptophan–kynurenine metabolism (*AFMID*) [62], lipid peroxidation (CYB5R1) [63], and tumor progression (*CCDC34*) [64–66]. Collectively, these findings suggest that the anti-leukemic activity of withaferin A is not mediated through a single molecular target but rather involves coordinated modulation of multiple biological processes that ultimately contribute to growth inhibition and apoptosis. Consistent with previous reports showing that DNMT1 inhibition-induced hypomethylation suppresses migration, invasion, drug resistance, and promotes programmed cell death [67–69], our findings suggest that withaferin A (**1**) may suppress leukemia progression through DNMT1-dependent DNA hypomethylation. Nevertheless, the present study has certain limitations that warrant further investigation. One limitation of this study is that the methylation-sensitive CMV-luc reporter assay was performed in KG-1 cells rather than in CCRF-CEM cells. This was because this experiment relies on a previously established stable CMV-luc reporter cell line, in which the CMV promoter is maintained in a partially methylated (~50%) form. This specialized reporter system requires stable integration and long-term maintenance of the methylation status and therefore cannot be readily reproduced in CCRF-CEM cells within a short timeframe. Importantly, the purpose of the CMV-luc assay was to utilize a validated methylation-sensitive reporter system to determine whether withaferin A is capable of reversing methylation-dependent transcriptional silencing. Future studies using methylation-sensitive reporter systems in T-ALL cells will be valuable to further validate the epigenetic effects of withaferin A. While our findings indicate that withaferin A (**1**) affects global DNA methylation through DNMT1 inhibition, it remains unclear how this mechanism specifically regulates target gene expression. The downregulation of genes such as *ITPKB, RAD21, PRR11*, and *CCDC34* may be attributable to demethylation occurring within gene bodies or enhancer/silencer regions. Alternatively, DNMT1 might act as a transcriptional activator for these genes, or it may play a role in maintaining their expression at the post-transcriptional level.

In summary, the findings demonstrate that withaferin A (**1**) is the most potent compound among its analogs, exhibiting the strongest inhibitory effects on leukemia cells. Withaferin A reduces DNA methylation levels and regulates the expression of multiple target genes, including *P21, ITPKB, RAD21, PRR11, AFMID, CCDC34*, and *CYB5R1*. These molecular events lead to apoptosis induction, G2/M cell-cycle arrest, suppression of cell migration, and inhibition of microtubule polymerization. This study systematically elucidates the functional network of withaferin A in T-ALL treatment. It reveals its mechanism of action through DNMT1-mediated promoter hypomethylation and transcriptional regulation of key target genes, providing new strategies for further exploration of the pharmacological mechanisms of withaferin A (**1**).

## Materials and methods

### Compounds

Withaferin A (**1**) was purchased from MedChem (Monmouth Junction, USA; Cat. No. HY-N2065). The compounds 4α-withaferin A diacetate (**2**), withaferin A diacetate (**3**), 27-deoxywithaferin A (**4**), 2,3-dihydrowithaferin A 3β-*O*-sulfate-27-*O*-acetate (**5**), 4α-withaferin A (**6**), and 2,3-dihydrowithaferin A 3β-*O*-sulfate (**7**) were provided by one of us (A. A. L. G.). The chemical structures of these withaferin derivatives are shown in Fig. 1. All compounds were dissolved in dimethyl sulfoxide (DMSO) to prepare 20 mM stock solutions and stored at −20 °C.

### Cell culture

Cell lines used in this study have been previously described [70]. CCRF-CEM (p53 mutant) (https://depmap.org/portal/cell_line/ACH-001738?tab=overview) and CEM/ADR5000 cells were cultured in RPMI 1640 medium (Cat. No. 11875093, Gibco, Carlsbad, CA, USA) supplemented with 10% fetal bovine serum (FBS; Cat. No. A5256701, Gibco, USA) and 1% penicillin-streptomycin (Cat. No. A5256701, Gibco, USA). Human bone osteosarcoma U2OS cells (p53 WT) [71] stably expressing α-tubulin-GFP (from Joachim Hehl, Light Microscopy Center, ETH Zurich), human embryonic kidney cells HEK293 and HEK293/ABCB5, breast cancer cells MDA-MB-231-pcDNA3 (p53 mutant) (https://depmap.org/portal/cell_line/ACH-000768?tab=overview) and MDA-MB-231-BCRP clone 23, wild-type colon cancer cells HCT116 (p53^+/+^) and HCT116 (p53^−/−^), as well as glioblastoma multiforme cells U87MG (p53 WT) [72] and U87MG.∆EGFR, were cultured in DMEM high-glucose medium (Cat. No. 11965092, Gibco, USA) supplemented with 10% FBS (Cat. No. A5256701, Gibco, USA) and 1% penicillin-streptomycin (Cat. No. 15140122, Gibco, USA). KG-1 cells (p53-mutant) (https://tp53.cancer.gov/results_cell_line_mutation) kindly provided by one of us (P. B. A.) were cultured in RPMI 1640 medium with GlutaMAX™ Supplement (Cat. No. 61870036, Gibco, USA), supplemented with 10% FBS and 1% geneticin (Cat. No. 10131035, Gibco, USA). In addition, resistant cell lines were maintained with continuous selection pressure: HCT116 (p53^−/−^) and U87MG.∆EGFR cells were cultured in the presence of 400 ng/mL geneticin, while MDA-MB-231-BCRP clone 23 cells were supplemented with 800 ng/mL geneticin every 2 weeks. All cells were incubated at 37 °C in a humidified atmosphere containing 5% CO₂. Cells in the logarithmic growth phase were used for all experiments. Cells were routinely tested and confirmed to be free of mycoplasma contamination.

### Resazurin reduction assay

The cytotoxicity of withaferin A (**1**) was assessed using a resazurin reduction assay. Suspension cells (10,000 cells/well) and adherent cells (5000 cells/well) were seeded in 100 µL of culture medium in 96-well plates. For suspension cells, the drug was added on the day of seeding, whereas for adherent cells, it was added after overnight attachment. Withaferin A (**1**) was serially diluted in 100 µL of culture medium to final concentrations of 100, 30, 10, 3, 1, 0.3, 0.1, 0.03, 0.01, and 0.003 μM. After 72 h of drug treatment, 20 µL of 0.01% resazurin solution (Cat. No. 62758-13-8, Sigma-Aldrich, St. Louis, Missouri, USA) were added to each well, followed by incubation at 37 °C for 4 h. Fluorescence was measured using an Infinite M200 Pro plate reader (Tecan, Crailsheim, Germany) at an excitation wavelength of 544 nm and an emission wavelength of 590 nm. Each experiment was independently repeated three times, with six technical replicates per concentration.

### Target gene screening method

Genes closely associated with T-ALL were identified using differential expression analysis and weighted gene co-expression network analysis (WGCNA) [73]. Datasets were selected from the GEO database based on the following criteria: organism (*Homo sapiens*), experiment type (expression profiling by array), and disease (leukemia). Then, two datasets were selected: GSE48558 (based on the GPL6244 platform) and GSE89565 (based on the GPL570 platform). From these datasets, samples of normal T cells and T-ALL were extracted, excluding those derived from the spleen or bone. This resulted in 17 normal T-cell samples and eight T-ALL samples in GSE48558, and 35 T-ALL samples in GSE89565. To minimize non-biological technical variation between datasets, the removeBatchEffect function from the limma package [74] in R (https://www.r-project.org/) was applied. Differentially expressed genes (DEGs) between the normal T cells and T-ALL groups were identified using the limma package with the following thresholds: *p* < 0.05 and |log₂FC | > 1.

Weighted Gene Co-expression Network Analysis (WGCNA) was then performed to identify gene modules with similar expression patterns and assess their association with clinical traits. From the GSE48558 and GSE89565 datasets, 60 samples were selected, and the top 5,000 genes with the highest median absolute deviation (MAD) were extracted to ensure expression variability. A soft-thresholding power of 5 was chosen to achieve a scale-free topology fit index (R² ≥ 0.85), and the adjacency matrix was computed using the absolute value of Pearson correlation coefficients raised to the power β. Based on this weighted network, a topological overlap matrix (TOM) was constructed to measure the interconnectivity of the genes, while the hierarchical clustering was used to group genes into modules. Dynamic tree cutting was applied with parameters deepSplit = 2 and minModuleSize = 30 to define initial gene modules. Then, modules with eigengene correlation > 0.75 were merged (mergeCutHeight = 0.25). Each module was assigned a unique color, while unassigned genes were grouped into the gray module. Correlation between module eigengenes and clinical traits (*e.g*., normal *vs*. T-ALL) was then computed and visualized using a labeled heatmap.

Additionally, the overall relationship between module membership (MM) and gene significance (GS) for genes within the blue module was evaluated. Module membership was calculated as the Pearson correlation between the expression profile of each gene and the module eigengene, while gene significance represented the correlation between individual gene expression and clinical trait. A scatter plot was used to illustrate the distribution pattern of MM and GS values, providing a visual representation of the relevance of the module to the trait of interest.

To identify genes differentially regulated by withaferin A (**1**) in CUTLL cells (a newly reported T-ALL cell line) [75], the GSE78188 dataset (platform: GPL10558) was downloaded from the GEO database. From the dataset, two groups – a withaferin A-treated group and a DMSO control group - were extracted, each consisting of three samples, and were subjected to differential expression analysis using the limma package in R with thresholds of *p *< 0.05 and |log₂FC | > 0.58.

### DNA methylation microarray analysis

Genome-wide DNA methylation datasets from GSE104359 (three wild-type samples and two DNMT1 siRNA samples) were analyzed using the ChAMP package [76, 77]. The workflow included data filtering, quality control, normalization, and identification of differentially methylated positions (DMPs). The raw β value matrix was normalized using the champ.norm() function. DMPs between wild-type and DNMT1 knockdown samples were identified using the champ.DMP() function with thresholds of |Δβ | ≥ 0.2 and FDR (adj.P.Val) < 0.05. Visualization of methylation patterns included: (i) Density plots: β values of significant DMPs were extracted from the normalized β value matrix and visualized with geom_density() in ggplot2, with the x-axis representing β values (0–1) and the y-axis indicating density; (ii) Heatmaps: the top 1000 hypermethylated probes and top 1000 hypomethylated probes (by absolute Δβ) were selected, and their β values were Z-score normalized before generating clustered heatmaps; iii) Genomic annotation analysis: The distribution of DMPs was calculated across CpG island regions (island, shore, shelf, open sea) and genomic functional regions (TSS, UTR, exon, intron, intergenic regions, IGR).

### Functional enrichment analysis

Gene Ontology (GO) analysis (covering biological processes, cellular components, and molecular functions) and Kyoto Encyclopedia of Genes and Genomes (KEGG) pathway enrichment analysis were conducted using the clusterProfiler package in R (https://www.r-project.org/) [78].

### Single-cell RNA-seq data processing and analysis

To investigate the cell types potentially targeted by withaferin A (**1**) in T-cell acute lymphoblastic leukemia (T-ALL), the single-cell transcriptomic dataset GSE227122. Only ten diagnostic T-ALL samples were included in the analysis, while post-treatment and relapsed samples were excluded to avoid therapy-related transcriptional alterations. Cells were initially filtered based on quality control metrics: cells expressing fewer than 300 or more than 4450 genes, with total Unique Molecular Identifiers (UMI) counts below 600 or above 22,000, or mitochondrial gene content exceeding 20%, and hemoglobin gene content exceeding 1% were excluded.

Following quality control, gene expression counts were normalized using the LogNormalize method (scale factor = 10,000), and 2000 highly variable genes were identified using the variance stabilizing transformation (vst) method. To minimize the effect of cell cycle heterogeneity, S.Score and G2M.Score were regressed during data scaling using the ScaleData function.

Principal component analysis (PCA) was conducted for dimensionality reduction, and the top 41 principal components (PCs) explaining > 90% variance were retained. Batch effects among different samples were corrected using the Harmony algorithm with orig.ident as the batch variable.

Cells were clustered by first constructing a shared nearest neighbor (SNN) graph with FindNeighbors, then using FindClusters at multiple resolutions (0.1 to 2.0). Clustering stability across resolutions was evaluated using the clustree package [79]. A final resolution of 0.5 was selected. Two-dimensional visualization was performed via t-SNE, based on PCA embeddings.

Putative cell types were annotated using the SingleR algorithm with the Blueprint-ENCODE reference dataset. Predicted labels were added to the Seurat object metadata. Cell type annotation was further refined using canonical marker genes from the CellMarker database (http://117.50.127.228/CellMarker/) and differentially expressed genes identified by FindAllMarkers (log2FC > 1, min. pct > 0.3).

Differential gene expression analysis was performed across clusters and cell types. The top five marker genes for each cluster were visualized using DotPlot. Finally, cell-type proportions across samples were visualized using stacked bar plots generated via ggplot2.

To evaluate the activity of specific core gene sets at the single-cell level, AUCell analysis (https://bioconductor.org/packages/release/bioc/html/AUCell.html) was performed using a Seurat object. A normalized expression matrix was first extracted using the AUCell package, and genes with zero total expression were filtered out. The core list of differentially expressed genes was then used to construct a single GeneSet object. Gene rankings were generated for each cell using AUCell_buildRankings(), followed by calculation of gene set activity scores (AUC values) with AUCell_calcAUC(). The resulting AUC scores were added to the meta.data slot of the Seurat object. For visualization, tSNE coordinates and corresponding AUC scores were extracted and combined with cell-type annotations. A two-dimensional embedding plot was generated using ggplot2 and ggrepel, to illustrate the distribution of gene-set activity across different cell subpopulations.

### RNA extraction and microarray hybridization

One million CCRF-CEM cells were seeded per well in six-well plates and treated for 24 h with either DMSO (control) or withaferin A (**1**) at its IC_50_ concentration. Total RNA was extracted using the InviTrap^®^ Spin Universal RNA Mini Kit (Invitek Molecular, Berlin, Germany). The RNA concentrations were measured with a NanoDrop 1000 spectrophotometer (Thermo Fisher Scientific, Waltham, MA, USA). For each sample, 1 μg of total RNA (50 ng/μL, 20 μL) was used for microarray hybridization. RNA quality control, probe labeling, hybridization, scanning, and initial data analysis were performed at the Genomics and Proteomics Core Facility of the German Cancer Research Center (DKFZ, Heidelberg, Germany). The microarray analysis was conducted using the Affymetrix Clariom S Assay HT, Human Platform [GPL24324], which includes the Pico Assay. Raw expression data were normalized using quantile normalization.

The resulting expression matrix was uploaded to the Chipster™ platform (https://chipster.csc.fi) for differential expression analysis, including quality control, filtering, and statistical testing, following procedures as previously described [80].

### Ingenuity pathway analysis (IPA)

Differentially expressed genes identified through the Chipster™ platform were imported into Ingenuity Pathway Analysis (IPA^®^, Qiagen, Redwood City, CA, USA) for further analysis. The core analysis was performed using default settings to explore significantly affected canonical pathways, biological functions, upstream regulators, and gene interaction networks. A *p*-value *p*-value threshold of < 0.05 was applied for statistical significance.

### Quantitative real-time reverse transcription PCR (qRT-PCR)

Reverse transcription of RNA was performed using the LunaScript^®^ RT SuperMix Kit (Cat. No. NEB #E3010, New England BioLabs, Frankfurt, Germany) according to the manufacturer’s instructions. Primers were designed using Primer-BLAST (https://www.ncbi.nlm.nih.gov/tools/primer-blast/) and synthesized by Eurofins Genomics (Ebersberg, Germany). Quantitative PCR reactions were set up following the instructions of the Hot Start Taq Probe qPCR Mix (No ROX) (Cat. No. 27488, Axon Labortechnik, Kaiserslautern, Germany). Then, qRT-PCR was carried out using the CFX384 Touch Real-Time PCR Detection System (Bio-Rad, Munich, Germany). CFX Manager Software (version 3.1) was used to determine the Cq (quantification cycle) values. The qPCR cycling conditions were set according to previously published protocols [81]. *GAPDH* was used as an internal control. Relative gene expression levels were calculated using the 2^−ΔΔCt method. Primer sequences used in this study are listed in Table 1.

### DNMT1 activity/inhibitor screening assay

The DNMT1 activity assay was performed in vitro following the manufacturer’s instructions of the EpiQuik™ DNA Methyltransferase 1 Activity / Inhibitor Screening Assay Core Kit (Cat. No. P-3006A, Epigentek). Reactions were set up in an eight-well substrate-coated strip, including blank, DMSO control, and compound-treated wells. Blank wells: 27 μL DNMT Assay Buffer and 3 μL diluted Adomet were added. DMSO control and compound-treated wells: 23 μL DNMT Assay Buffer, 3 μL diluted Adomet, 1 μL DNMT1 enzyme (E15000-1, Epigentek), and 3 μL of the diluted test compound (or an equal volume of diluted DMSO for control) were added. All reactions were incubated at 37 °C for 1.5 h. After incubation, the subsequent color developer and stop reactions were applied according to the kit protocol. Absorbance (OD) was measured at 450 nm using an Infinite M200 Pro plate reader (Tecan, Crailsheim, Germany). DNMT1 activity was calculated using the following formula:

$${{\rm{DNMT}}}\; {{\rm{activity}}}({{\rm{OD}}}/{{\rm{h}}}/{{\upmu}} {{\rm{g}}})=({{\rm{no}}}\; {{\rm{in}}}{{\rm{h}}}{{\rm{ibitor}}}\; {{\rm{OD}}}{-}{{\rm{blank}}}\; {{\rm{OD}}}){{\rm{x}}} \\ 1000/({{\rm{DNMT}}}\,1\,{{\rm{amount}}}\left({{\rm{ng}}}\right){{\rm{added}}}\; {{\rm{in}}}\; {{\rm{t}}}{{\rm{h}}}{{\rm{e}}}\; {{\rm{reaction}}}\times 1.5{{\rm{h}}})$$

### Wound healing assay

The wound healing assay was carried out using U2OS cells stably expressing α-tubulin-GFP and HCT116 p53^–/–^ cells. Cells were seeded in Culture-Insert 2 Well 24 (Cat. No. 80242, ibidi, Germany) to establish the wound model, following the manufacturer’s instructions. Cells were maintained in culture medium containing 1% serum. DMSO served as the control group, while withaferin A (**1**) was applied at concentrations of 0.5 × IC₅₀ and IC₅₀. Images were captured at 0 h and 24 h using a JuLI™ Br Live Cell Movie Analyzer (NanoEnTek Inc., Seoul, Republic of Korea) at 1× magnification. All experiments were independently repeated three times.

### Apoptosis assay

CCRF-CEM cells in logarithmic growth phase (1 × 10⁶ cells) were seeded into six-well plates and treated with DMSO (control) or withaferin A (0.5 × IC₅₀, IC₅₀, and 2 × IC₅₀) for 48 h. Cells were harvested, and apoptosis was assessed using the Annexin V-FITC/PI double staining kit (Invitrogen, Life Technologies GmbH, Darmstadt, Germany) according to the manufacturer’s protocol. Briefly, cells were resuspended in cold PBS, centrifuged, and washed with 1 × binding buffer, followed by incubation with Annexin V-FITC for 15 min at room temperature in the dark. Propidium iodide (PI) was then added before analysis on a flow cytometer (BD Accuri™ C6, BD Biosciences, Franklin Lakes, NJ, USA). Experiments were independently repeated three times, and data were analyzed using FlowJo software (version 10.10.0, Celeza, Olten, Switzerland).

### Cell cycle analysis

CCRF-CEM cells in logarithmic growth phase (1 × 10⁶ cells) were seeded into six-well plates. Cells were treated with DMSO (negative control), withaferin A (0.5 × IC₅₀, IC₅₀, and 2 × IC₅₀), or cisplatin (5 μM, positive control) for 48 h. After treatment, cells were collected, washed three times with cold PBS, and fixed in 75% ice-cold ethanol at −20 °C overnight. On the following day, the cells were centrifuged, washed three times with PBS, and incubated with 500 μL PBS containing RNase A (Roche Diagnostics, Mannheim, Germany) for 30 min at room temperature. Subsequently, cells were stained with propidium iodide (PI, 50 μg/mL; Sigma-Aldrich, Germany) for 15 min in the dark at room temperature. Cell cycle distribution was analyzed using a BD Accuri™ C6 flow cytometer, with three independent replicates, and data were processed using FlowJo software.

### Luciferase reporter assay

The luciferase reporter assay was performed using the KG-1 cell line, which was stably transfected with a construct encoding the firefly luciferase gene under the control of the cytomegalovirus (CMV) promoter. The CMV promoter is sensitive to DNA methylation, and the vector construction of this cell line has been described previously [82]. Cells were seeded into 96-well plates at a density of approximately 2 × 10⁵ cells per well. DMSO was used as the negative control, and withaferin A (**1**) was applied at concentrations of 4 μM, 6 μM, and 8 μM. After 24 h of incubation, 10 µL of Luciferase Assay Substrate (Cat. No. E6110, Promega, Madison, USA) were added to each well. Luciferase activity was measured using an Infinite® M2000 Pro microplate reader (Tecan, Crailsheim, Germany). All experiments were performed in triplicate.

### Immunofluorescence staining

Approximately 3 × 10⁴ HCT116 p53^+/+^ cells were seeded per well on eight-well chamber slides (Cat. No. 80841, ibidi, Fitchburg, WI, USA). Cells were treated with DMSO, withaferin A (IC₅₀ and 2 × IC₅₀), paclitaxel (5 μM), or vincristine (5 μM) for 24 h. After treatment, the cells were washed three times with PBS, then fixed with 4% paraformaldehyde for 15 min, followed by another three washes with PBS. Cells were permeabilized with 0.1% Triton X-100 for 10 min and blocked with 5% bovine serum for 1 h. Primary antibody against β-tubulin (1:50, Cat. No. 2146, Cell Signaling Technology, Danvers, MA, USA) was applied and incubated overnight at 4 °C. The next day, after equilibration to room temperature, cells were washed three times with PBS and incubated with Alexa Fluor 488–conjugated anti-rabbit secondary antibody (1:100, Cat. No.4412, Cell Signaling Technology, Danvers, MA, USA) at room temperature for 1 h. Nuclei were counterstained with 1 μg/mL DAPI (Sigma-Aldrich, St. Louis, MO, USA) for 15 min after three PBS washes. Finally, sample cells were mounted with Fluoromount-G^®^ (SouthernBiotech, Birmingham, AL, USA) and imaged using an AF7000 Widefield Fluorescence Microscope at 40× magnification.

### Zebrafish preparation and determination of the maximum tolerated concentrations (MTC)of withaferin A (**1**)

Adult zebrafish were supplied by the company’s fish breeding center, Institute of Hydrobiology, China Academy of Science (Wuhan, China), and the Association for Assessment and Accreditation of Laboratory Animal Care International approved the use of the animals in this experiment (Accreditation No.: IACUC-2025-202509030001-01). Zebrafish were reared in aquarium water maintained at 28 °C (water composition: 200 mg of instant sea salt added to 1 L of reverse osmosis water; conductivity 450–550 μS/cm; hardness: 50–100 mg/L CaCO₃). To select the optimum dose of withaferin A (**1**), a wild-type AB strain zebrafish at 3 d post-fertilization (3 dpf) were randomly selected and incubated in a six-well plate, with 30 fish per well. DMSO was used to dissolve withaferin A (**1**), and the final concentrations (3.12, 6.25, 12.5, 25.0, and 50.0 μM) were administered in water. After incubation at 35 °C for 2 d, the MTC of the samples on normal zebrafish was determined. Withaferin A (**1**) did not induce any observable abnormal phenotypes within the concentration range of 0–3.12 μM. Therefore, 3.12 μM was considered the highest concentration corresponding to the no-observed-adverse-effect level.

### Xenograft anti-cancer experiment

To prepare a tumor xenograft model in zebrafish, CCRF-CEM cells labeled with CM-DiI (red fluorescence) were microinjected into the yolk sac of 2-dpf zebrafish larvae with approximately 200 cells per fish. Only zebrafish larvae showing successful and comparable tumor engraftment at 3 dpf were included in the subsequent analysis. Eligible zebrafish larvae were randomly assigned to the different treatment groups before drug administration. Blinding was not performed during treatment allocation. Then, withaferin A (**1**) was administered in water (0.781, 1.56, and 3.12 μM), with cisplatin (15.0 μg/mL) serving as the positive control, and an untreated A model was also established as a negative control. After tumor growth for 2 d of incubation at 35 °C,13 zebrafish were randomly selected from each experimental group and photographed under a fluorescence microscope (AZ100, Nikon, Tokyo, Japan). The fluorescence intensity (Fi) of CCRF-CEM cell mass in each fish was collected using NIS-Elements D 3.20 advanced image processing software. The antitumor efficacy was calculated as: inhibitory rate (%) = [1–(Fi of treated group/Fi of NC group)] × 100%.

### Colony formation assay

Cells were seeded into f 6-well plates with cell density of 2 × 10^3^ cells/ well. They were treated with withaferin A (**1**) for 72 h, after which the drug-containing medium was removed and replaced with fresh medium before colony formation. After 7 d of incubation, colonies were fixed with 4% paraformaldehyde, stained with crystal violet (MedChem Cat. No. HY-B0324A) according to the manufacturer’s instructions (https://file.medchemexpress.com/batch_PDF/HY-B0324A/Crystal-Violet-DataSheet-MedChemExpress.pdf) and counted.

### Live/dead cell staining

Cell viability was assessed using the LIVE/DEAD™ Fixable Dead Cell Stain Kit (Cat. No. L34969, Thermo Fisher Scientific, Waltham, MA, USA) according to the manufacturer’s instructions (https://documents.thermofisher.com/TFS-Assets/LSG/manuals/live_dead_fixable_dead_cell_stains_man.pdf). The proportions of live and dead cells were determined using a BD Accuri™ C6 flow cytometer. Three independent experiments were performed, and the data were analyzed using FlowJo software.

### Western blot

Western blotting was performed using rabbit anti-p53 (1:1000, Cat. No. 2527), rabbit anti-p21 (1:500, Cat. No. 2947), rabbit anti-GAPDH (1:1000, Cat. No. 2118), and HRP-conjugated anti-rabbit IgG secondary antibody (1:1000, Cat. No. 7074) (all antibodies are from Cell Signaling Technology, Danvers, MA, USA). All other experimental procedures were carried out as previously described [81].

### Statistical analysis

Statistical analyses were performed using GraphPad Prism 8 (GraphPad Software, USA). Data were first assessed for normality and homogeneity of variance. Comparisons among multiple groups were conducted using one-way ANOVA, whereas differences between two groups were evaluated using a two-tailed *t*-test. Data are expressed as the mean ± standard deviation (SD) from at least three independent experiments. Statistical significance was defined as *p* < 0.05 (*p < 0.05, **p < 0.01, ***p < 0.001).

## Supplementary information

Supplementary Information

Uncropped original Western blot data

## Data Availability

Data that support the findings of this study are available from the corresponding author upon reasonable request.
