# Supplementary material for: Withaferin A suppresses malignant progression of leukemia by reducing promoter methylation levels
Source: Cell Death Discov. 2026 Nov 13;12:457. doi: 10.1038/s41420-026-03435-x (PMC13768349; doi:10.1038/s41420-026-03435-x)
Supplement: Supplementary file 1 [file 41420_2026_3435_MOESM1_ESM.pdf]

Supplementary Information

Figure S1

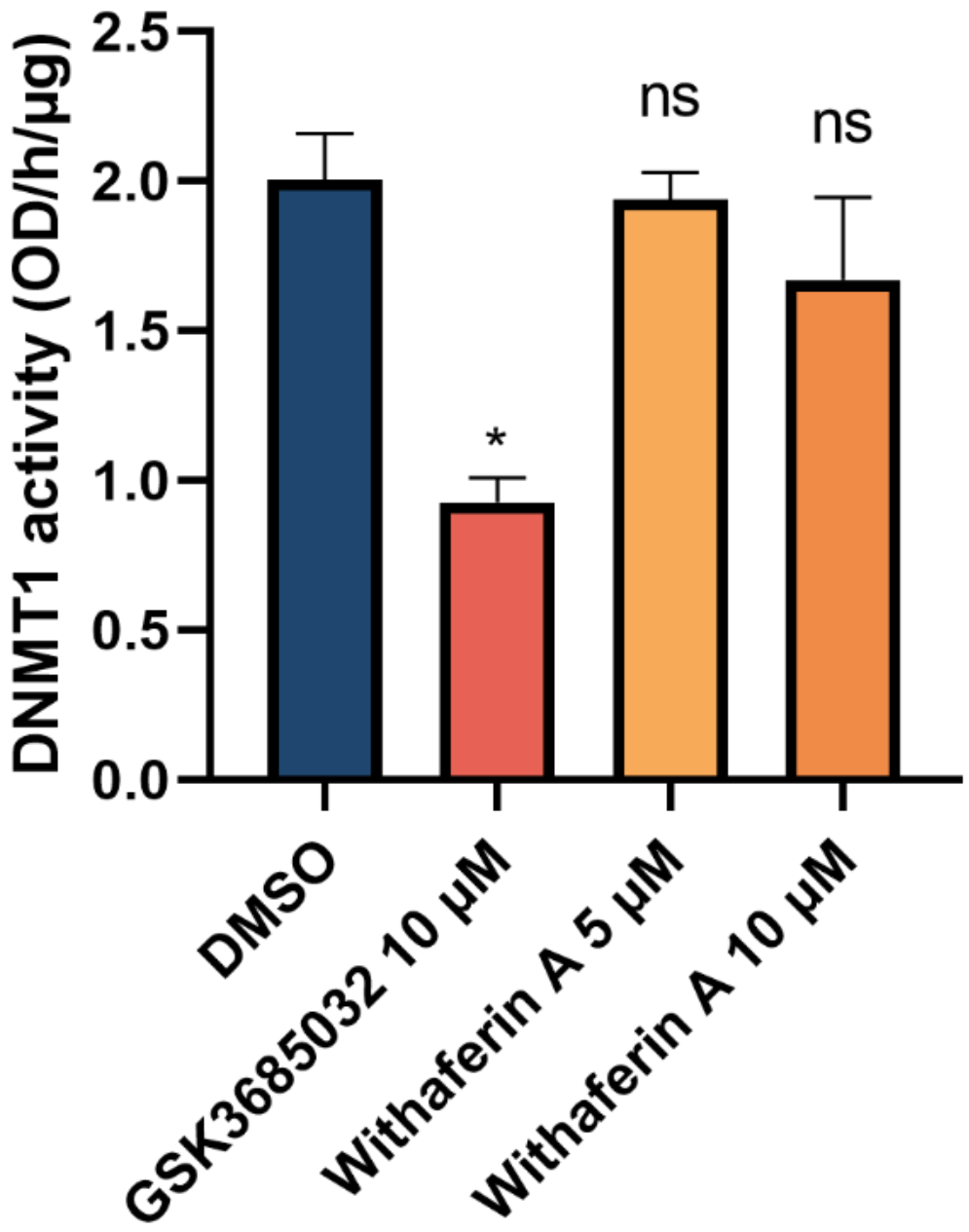

Figure S1. DNMT1 activity assay shows no significant difference following withaferin A treatment compared with the DMSO control (mean ± SD, n = 3, ns, $p > 0.05$).

Figure S2

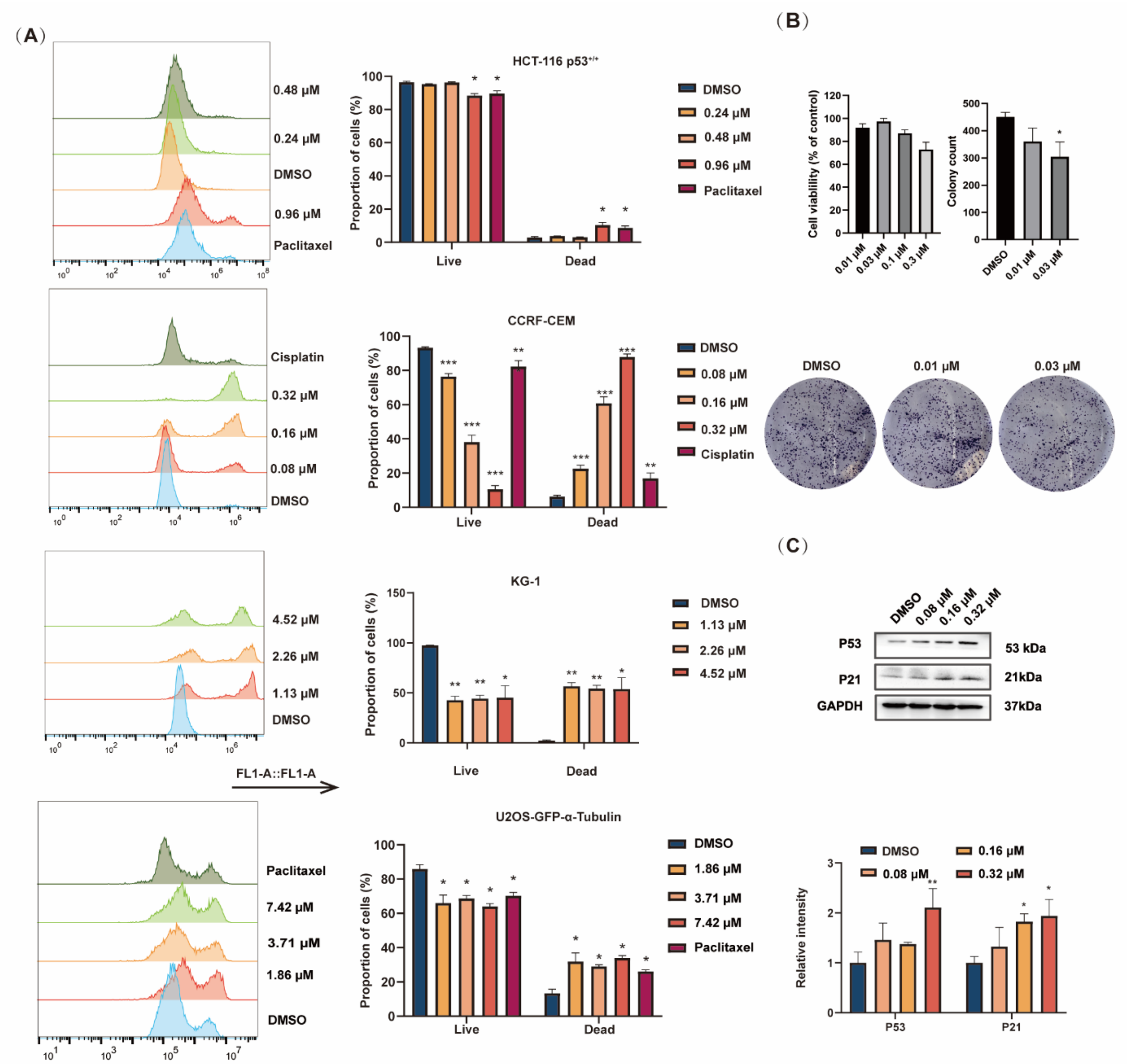

Figure S2. Cytotoxic and subtoxic effects of withaferin A (**1**) and regulation of p53 and p21 protein expression. (A) Cytotoxicity of withaferin A (**1**) in four cancer cell lines was evaluated by LIVE/DEAD staining. (B) Subtoxic effects of withaferin A (**1**) were evaluated by colony formation assays. (C) Effects of withaferin A (**1**) on p53 and p21 protein expression in CCRF-CEM cells were determined by western blot analysis (mean ± SD, n=3). *, $p < 0.05$; **, $p < 0.01$; ***, $p < 0.001$.

Figure S3

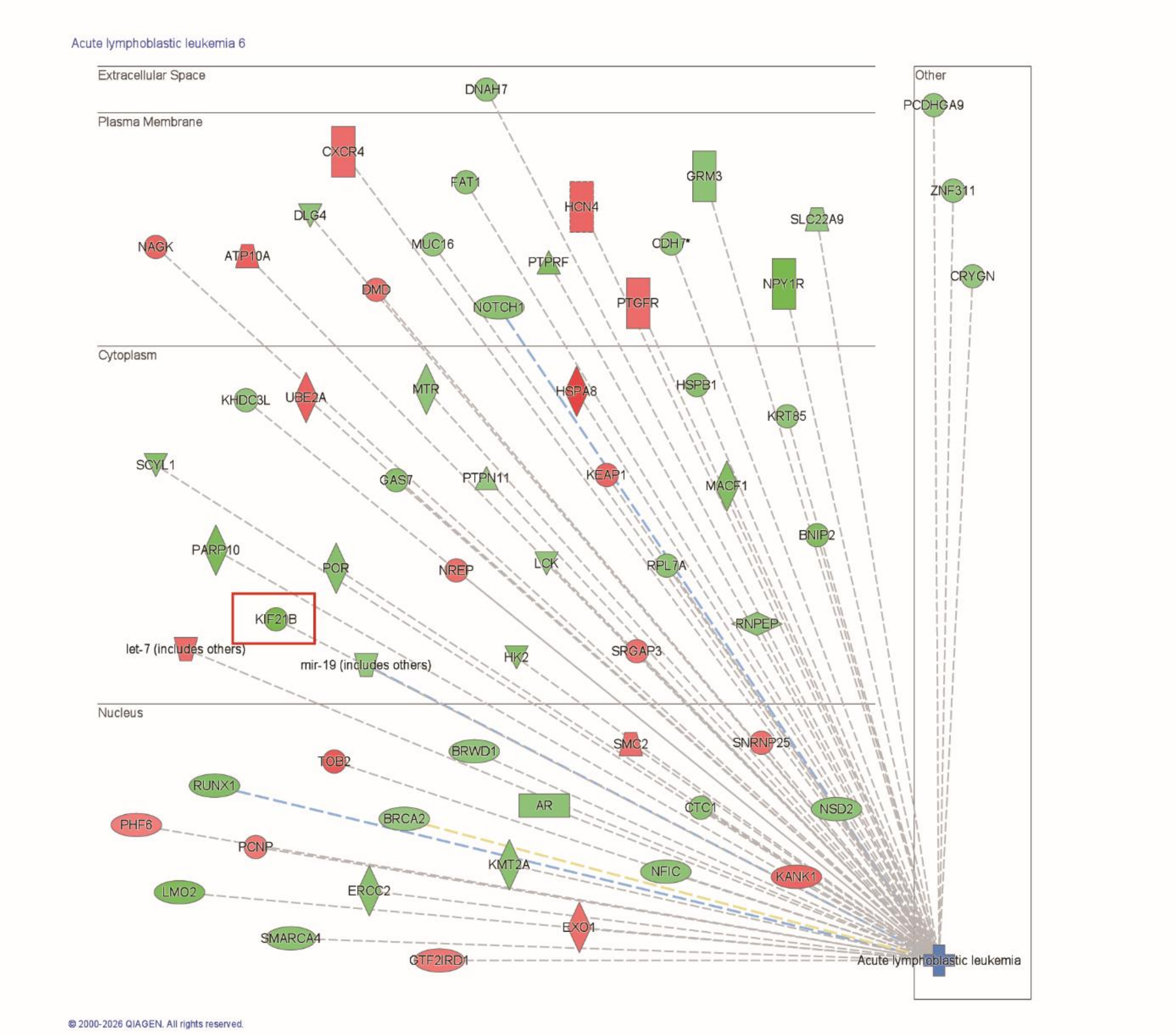

© 2000-2026 QIAGEN. All rights reserved.

Figure S4

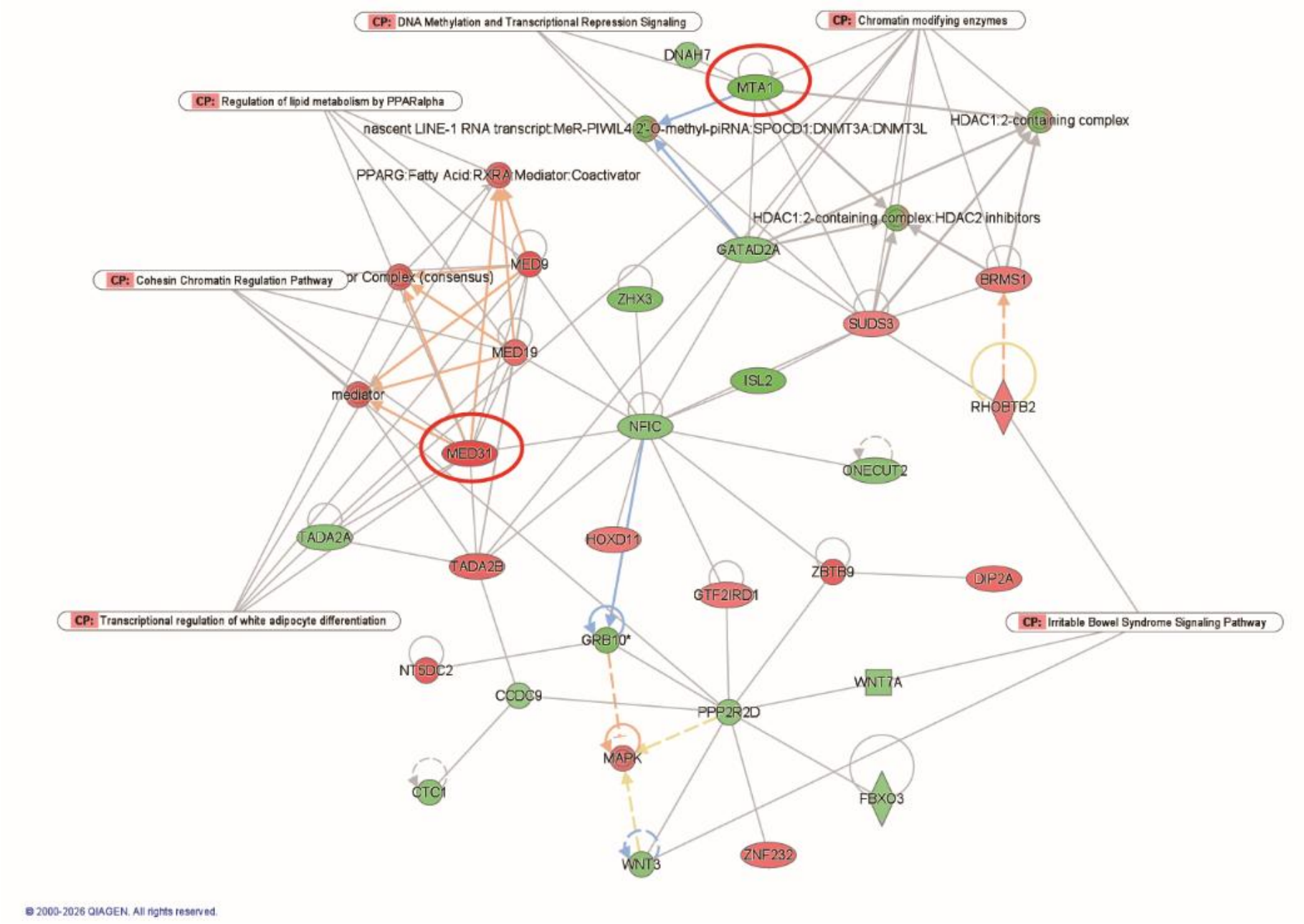

© 2000-2026 QIAGEN. All rights reserved.

Figure S3 and S4. IPA analysis of gene regulatory networks and biological functions in CCRF-CEM cells following withaferin A (**1**) treatment.
