# Supplementary material for: Withaferin A suppresses malignant progression of leukemia by reducing promoter methylation levels
Source: Cell Death Discov. 2026 Nov 13;12:457. doi: 10.1038/s41420-026-03435-x (PMC13768349; doi:10.1038/s41420-026-03435-x)
Supplement: Supplementary file 2 [file 41420_2026_3435_MOESM2_ESM.pdf]

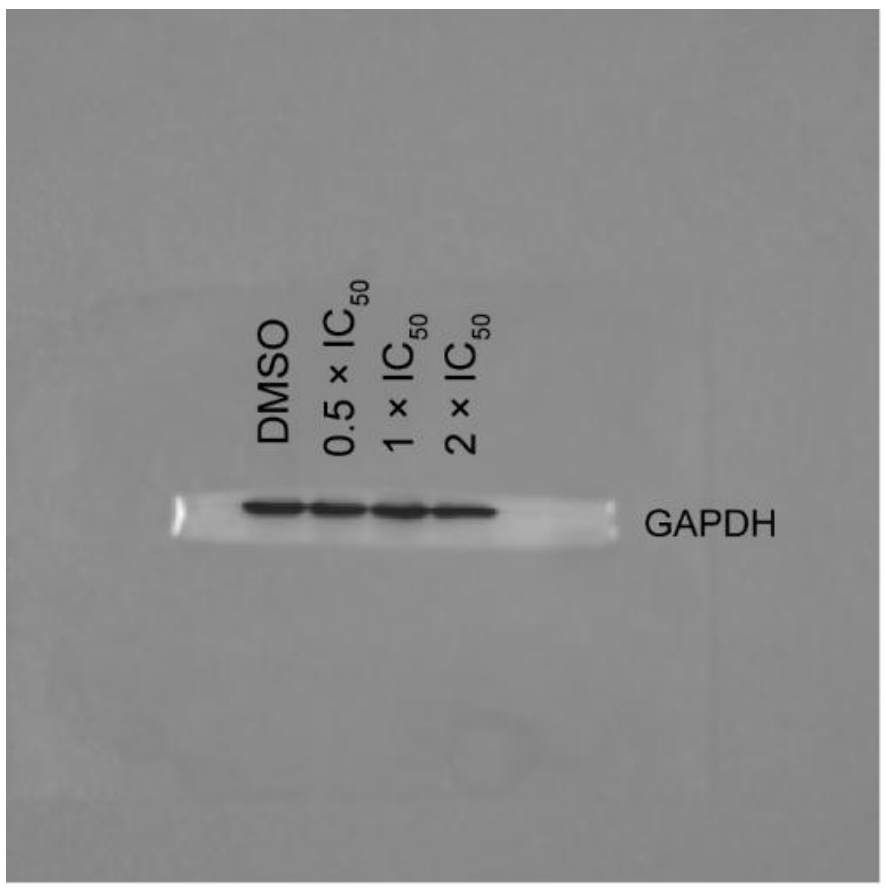
DMSO
0.5 × IC_{50}
1 × IC_{50}
2 × IC_{50}
GAPDH

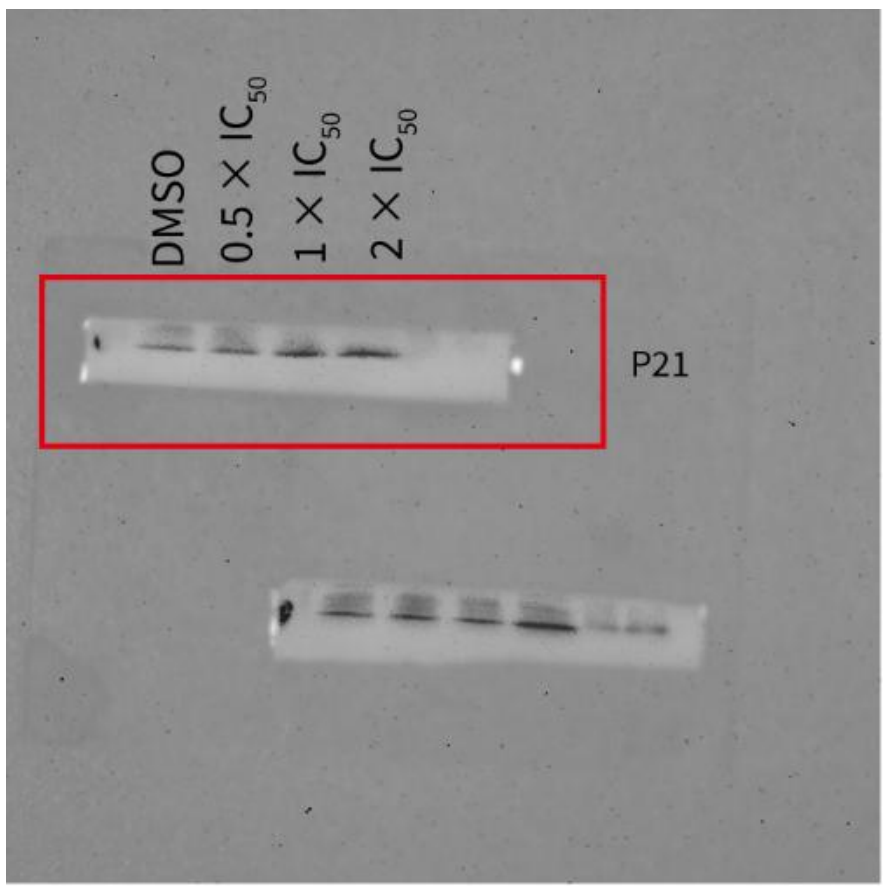
DMSO
0.5 × IC_{50}
1 × IC_{50}
2 × IC_{50}
P21

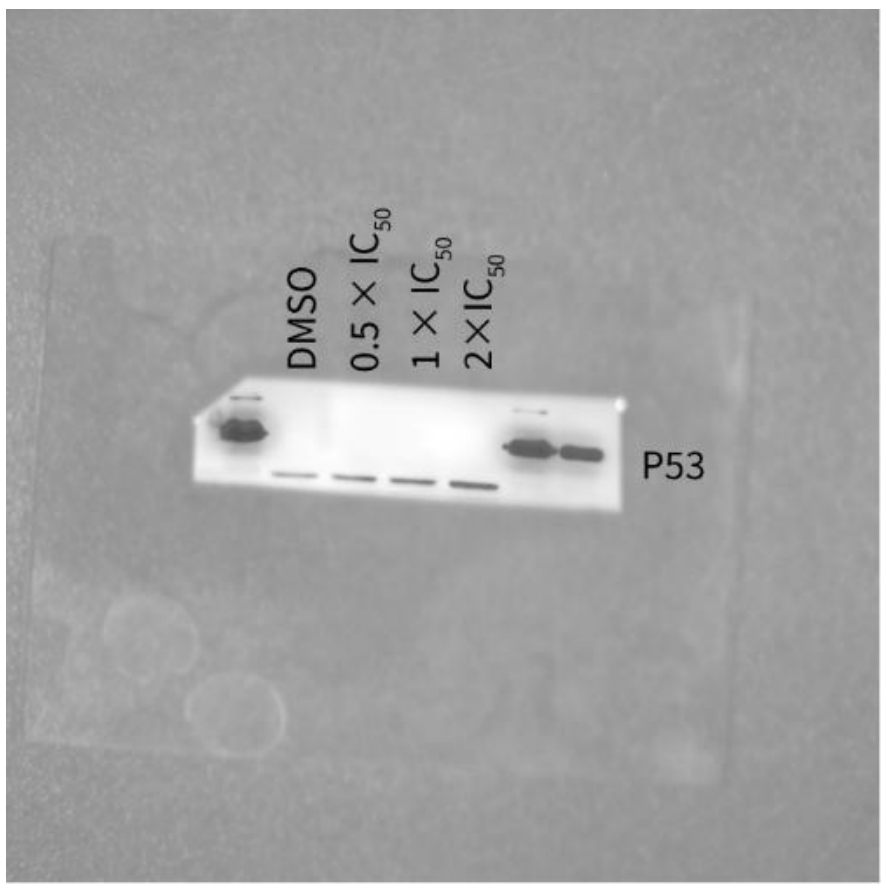
DMSO
0.5 × IC_{50}
1 × IC_{50}
2× IC_{50}
P53
